# HIF-1α-induced expression of m6A reader YTHDF1 drives hypoxia-induced autophagy and malignancy of hepatocellular carcinoma by promoting ATG2A and ATG14 translation

**DOI:** 10.1038/s41392-020-00453-8

**Published:** 2021-02-23

**Authors:** Qing Li, Yong Ni, Liren Zhang, Runqiu Jiang, Jing Xu, Hong Yang, Yuanchang Hu, Jiannan Qiu, Liyong Pu, Jinhai Tang, Xuehao Wang

**Affiliations:** 1 Hepatobiliary Center, The First Affiliated Hospital of Nanjing Medical University; Key Laboratory of Liver Transplantation, Chinese Academy of Medical Sciences; NHC Key Laboratory of Living Donor Liver Transplantation (Nanjing Medical University), Nanjing, Jiangsu Province China; 2 School of Medicine, Southeast University, Nanjing, China; 3 Department of Hepatopancreatobiliary Surgery, Shenzhen Second People’s Hospital, The First Affiliated Hospital of Shenzhen University, Shenzhen, Guangdong China; 4 Department of Hepatobiliary Surgery, The Affiliated Drum Tower Hospital of Nanjing University Medical School, Nanjing, Jiangsu Province People’s Republic of China; 5 Medical School of Nanjing University, Nanjing, Jiangsu China; 6 Department of Oncology, The First Affiliated Hospital of Nanjing Medical University, Nanjing, Jiangsu Province China; 7 Department of Immunology, Key Laboratory of Immune Microenvironment and Disease, Nanjing Medical University, Nanjing, Jiangsu Province China; 8 Department of General Surgery, The First Affiliated Hospital of Nanjing Medical University, Nanjing, Jiangsu Province China

**Keywords:** Cancer, Prognostic markers

## Abstract

N6-methyladenosine (m6A), and its reader protein YTHDF1, play a pivotal role in human tumorigenesis by affecting nearly every stage of RNA metabolism. Autophagy activation is one of the ways by which cancer cells survive hypoxia. However, the possible involvement of m6A modification of mRNA in hypoxia-induced autophagy was unexplored in human hepatocellular carcinoma (HCC). In this study, specific variations in YTHDF1 expression were detected in YTHDF1-overexpressing, -knockout, and -knockdown HCC cells, HCC organoids, and HCC patient-derived xenograft (PDX) murine models. YTHDF1 expression and hypoxia-induced autophagy were significantly correlated in vitro; significant overexpression of YTHDF1 in HCC tissues was associated with poor prognosis. Multivariate cox regression analysis identified YTHDF1 expression as an independent prognostic factor in patients with HCC. Multiple HCC models confirmed that YTHDF1 deficiency inhibited HCC autophagy, growth, and metastasis. Luciferase reporter assays and chromatin immunoprecipitation demonstrated that HIF-1α regulated YTHDF1 transcription by directly binding to its promoter region under hypoxia. The results of methylated RNA immunoprecipitation sequencing, proteomics, and polysome profiling indicated that YTHDF1 contributed to the translation of autophagy-related genes ATG2A and ATG14 by binding to m6A-modified ATG2A and ATG14 mRNA, thus facilitating autophagy and autophagy-related malignancy of HCC. Taken together, HIF-1α-induced YTHDF1 expression was associated with hypoxia-induced autophagy and autophagy-related HCC progression via promoting translation of autophagy-related genes ATG2A and ATG14 in a m6A-dependent manner. Our findings suggest that YTHDF1 is a potential prognostic biomarker and therapeutic target for patients with HCC.

## Introduction

Hepatocellular carcinoma (HCC) is one of the most prevalent lethal tumors, with Chinese HCC patients accounting for 50% of global cases.^1,2^ Despite the tremendous progress in HCC treatment, therapeutic options remain limited.^3,4^ Only a small proportion of patients with HCC are eligible for curative treatments such as surgical resection, ablation, and liver transplantation. The majority of patients with HCC demonstrate postsurgical recurrence and metastasis, resulting in a poor 5-year overall survival rate.^5^ Hence, new therapeutic strategies are required to address these limitations.

Rapid growth, accompanied by insufficient blood supply, causes a hypoxic microenvironment in solid tumors,^6,7^ forcing tumor cells to adapt to hypoxic stress through invasion and metastasis. Previous studies have demonstrated that autophagy is a crucial adaptive feature that favors tumor survival under hypoxic conditions by clearing damaged organelles, regulating production of reactive oxygen species, and decreasing apoptosis.^8^ Recent research demonstrated that even in the absence of apoptosis, autophagy is essential and confers several advantages, including enhancing the viability of tumor cells under hypoxic stress.^9–11^ Activation of autophagy under hypoxic conditions has been shown to promote chemoresistance in a variety of tumors;^12^ thus autophagy suppression is a promising strategy in the development of antitumor therapies.^13^

Methylation modification resulting in N6-methyladenosine (m6A) is the most prevalent internal modification, ubiquitously occurring in eukaryotic mRNA.^14^ m6A modification can influence mRNA decay, splicing, transport, localization, and translation.^15,16^ In mammalian cells, m6A modification is dynamic, reversible, and catalyzed by m6A methyltransferases (METTL3, METTL14, and WTAP), also known as “writers”. m6A demethylases (FTO and ALKBH5), also known as “erasers”, are responsible for directly removing m6A modification of mRNA. Additionally, specific RNA-binding proteins (YTHDF1/2/3, IGF2BP1/2/3, eIF3, HNRNPA2B1, etc.), also known as “readers”, can bind to the m6A motif to influence RNA function, thus conferring specific phenotypic outcomes.^17,18^ Accumulating evidence has demonstrated the m6A modification is associated with various biological processes, including stem cell differentiation, tissue development, and tumor progression. A recent study determined that tumor hypoxia results in m6A epigenetic remodeling.^19^ Hypoxia-inducible factors (HIFs) play a vital role in m6A modification. However, the biological significance of m6A modification under hypoxia and its underlying regulatory mechanisms remain elusive, especially in human HCC hypoxia-induced autophagy.

Herein, we evaluated the oncogenic role of YTHDF1 in human HCC hypoxia-induced autophagy and autophagy-related HCC malignancy using multiple models, including HCC cells, HCC organoids, HCC patient-derived xenograft (PDX) murine models, and nude mice. The biological, mechanistic, and clinical implications of our study suggest a potential prognostic biomarker and therapeutic target for patients with HCC.

## Results

### YTHDF1 is closely correlated with hypoxia-induced autophagy

**Fig. 1:**
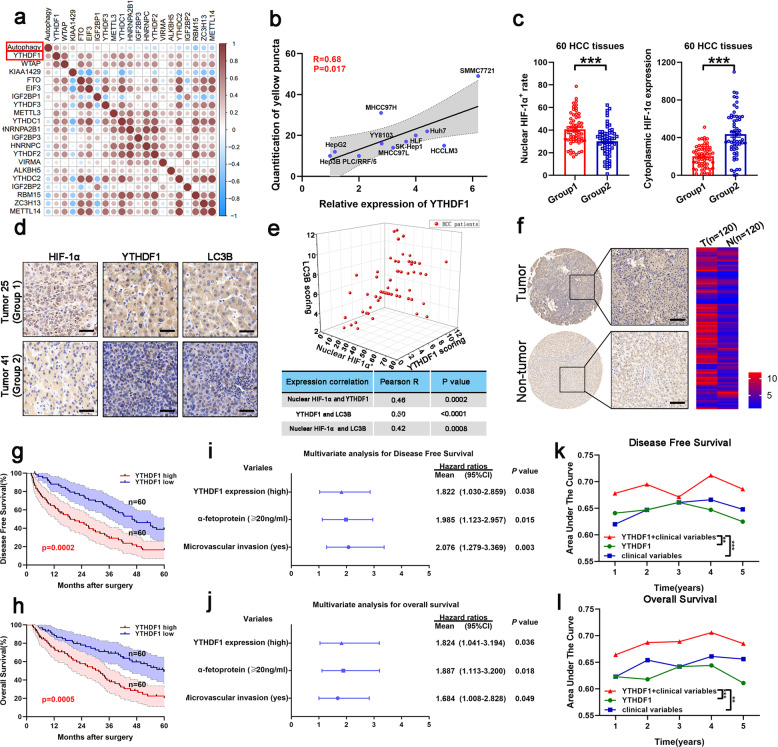
Increased YTHDF1 expression is associated with poor prognosis in patients with HCC. **a** Relationship between m6A gene expression and hypoxia-induced autophagy in HCC cell lines using a correlation matrix. **b** Relationship between YTHDF1 expression and hypoxia-induced autophagy in HCC cell lines. **c** Immunohistochemistry (IHC) staining of HIF-1α in human HCC tumors. **d** IHC staining of HIF-1α, YTHDF1, and LC3B in human HCC tumors. Scale bar, 100 µm. **e** Three-dimensional scatter plot of HIF-1α, YTHDF1, and LC3B in human HCC tumors. **f** Representative IHC images of YTHDF1 using HCC tissue microarray (TMA). Scale bar, 200 µm. **g**, **h** Kaplan–Meier analysis showing the disease-free survival and overall survival of HCC patients with diverse YTHDF1 expression. **I**, **j** Multivariate Cox regression analysis of disease-free survival and overall survival in patients with HCC. **k**, **l** Time-dependent receiver operating characteristic analysis for clinical risk score (microvascular invasion), YTHDF1 risk score, and combined YTHDF1 and clinical risk scores in patients with HCC. Error bars represent the mean ± SEM and the dots represent the value of each experiment; **P < 0.01, ***P < 0.001

Correlations between regulated m6A gene expression and autophagic flux in the HCC cell lines were determined (Supplementary Table S1), revealing that transcription of YTHDF1 was mostly correlated with autophagosome formation under hypoxic conditions (Fig. 1a, b and Supplementary Fig. S1a–c). Expression of HIF-1α, induced in response to hypoxic stress, was evaluated in 60 HCC samples to reveal two groups based on HIF-1α localization; HIF-1α was detected primarily in the nucleus in group 1, representing hypoxia within tumors,^20^ and in the cytoplasm in group 2 (Fig. 1c). The correlation between localization of HIF-1α and expression of YTHDF1 or LC3B in each group demonstrated that YTHDF1 was closely related to hypoxia and associated autophagy in human HCC (Fig. 1d, e).

### Increased YTHDF1 expression predicts poor prognosis of HCC patients

We employed bioinformatics-based screening to investigate the role of YTHDF1 in human HCC. YTHDF1 expression in HCC tissues was higher than that in normal tissues based on The Cancer Genome Atlas (TCGA) HCC database (Supplementary Fig. S2a). Further, expression of YTHDF1 was upregulated in HCC tissues compared with that in cirrhotic tissues based on the Gene Expression Omnibus (GEO) dataset (GSE63898) (Supplementary Fig. S2b).

We then evaluated YTHDF1 expression by quantitative reverse transcription PCR (qRT-PCR) in HCC and corresponding adjacent non-tumorous tissues, demonstrating that YTHDF1 expression was higher in HCC tissues than in non-tumorous tissues (Supplementary Fig. S2c). Increased YTHDF1 protein expression in HCC tissues was confirmed by western blotting of 12 randomly selected pairs of HCC and adjacent normal tissues (Supplementary Fig. S2d). Upregulation of YTHDF1 protein was further validated combining immunohistochemistry (IHC) staining with HCC tissue microarray (TMA) (Fig. 1f). Clinicopathological analysis demonstrated that YTHDF1 expression was closely correlated with tumor size, microvascular invasion, tumor-node-metastasis, and Edmonson stages (Supplementary Fig. S2e and Supplementary Table S2). Further, patients with high YTHDF1 expression had shorter disease-free survival (DFS) and poorer overall survival (OS) than those with low YTHDF1 expression (Fig. 1g, h). The bioinformatics tool GEPIA also validated that patients with HCC exhibiting increased YTHDF1 mRNA levels had worse DFS and OS (Supplementary Fig. S2f, g). Multivariate Cox regression analysis determined that high YTHDF1 expression was an independent prognostic factor for DFS (hazard ratio [HR] = 1.822, 95% confidence interval [CI]: 1.030–2.859; *P* = 0.038) and OS (HR = 1.824, 95% CI: 1.041–3.194; *P* = 0.036) in HCC patients (Fig. 1i, j and Supplementary Table S3). Time-dependent receiver operating characteristic curve analysis demonstrated that combining the clinical (microvascular invasion) and YTHDF1 risk scores contributed to improved predictive performance compared with the individual risk scores (Fig. 1k, l). Thus, the above results indicated that YTHDF1 was closely correlated with hypoxia-induced autophagy, and its upregulation predicted poor prognosis of patients with HCC.

### Hypoxia upregulated YTHDF1 expression in a HIF-1α-dependent manner

**Fig. 2:**
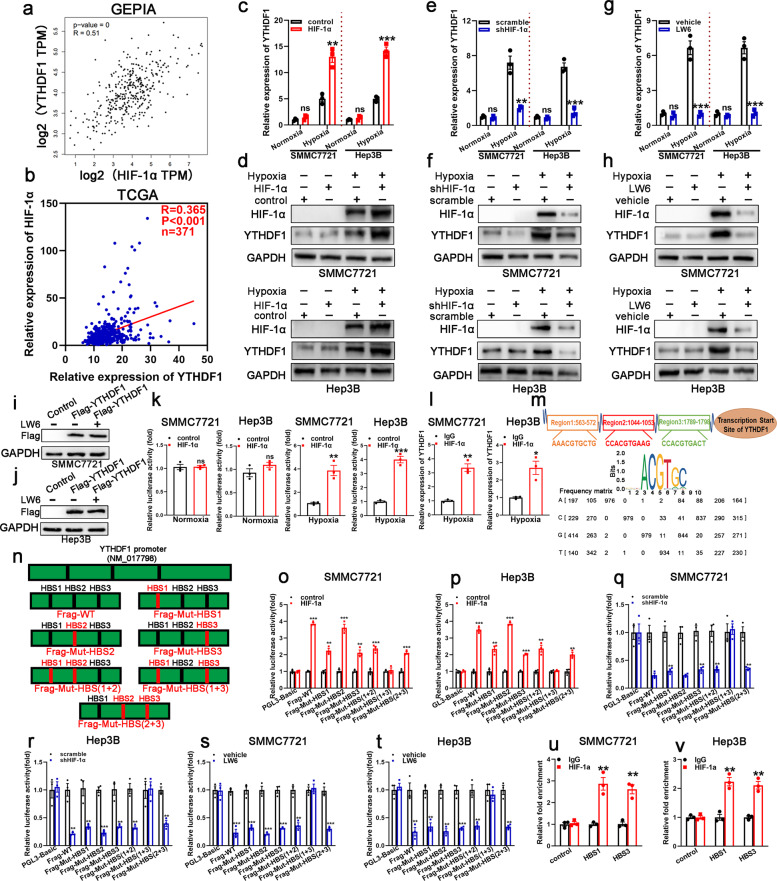
HIF-1α activates YTHDF1 transcription under hypoxia. **a** Correlation between mRNA expression of HIF-1α and YTHDF1 in the GEPIA database. **b** Correlation between mRNA expression of HIF-1-α and YTHDF1 in the TCGA database. **c**, **d** HCC cells with HIF-1α overexpression (HIF-1α) or not (control) were cultured under normoxia (20% O_2_) or hypoxia (1% O_2_), and expression of HIF-1α and YTHDF1 was detected using qRT-PCR (**c**) and western blotting (**d**), respectively. **e**, **f** HCC cells with HIF-1α knockdown (shHIF-1α) or not (scramble) were cultured under normoxia (20% O_2_) or hypoxia (1% O_2_), and expression of HIF-1α and YTHDF1 was detected using qRT-PCR (**e**) and western blotting (**f**), respectively. **g**, **h** HCC cells following LW6 treatment or not (vehicle) were cultured under normoxia (20% O_2_) or hypoxia (1% O_2_), and expression of HIF-1α and YTHDF1 was detected using qRT-PCR (**g**) and western blotting (**h**), respectively. **i**, **j** Western blotting of YTHDF1/FLAG levels treated with or without LW6. **k** Luciferase reporter assays were performed in SMMC7721 and Hep3B cells following HIF-1α overexpression under normoxia or hypoxia. **l** Chromatin immunoprecipitation (ChIP) assays in SMMC7721 and Hep3B cells to assess the binding of HIF-1α to the YTHDF1 promoter under hypoxia. **m** Putative HIF-1α-binding sites (HBS) within the genomic sequence adjacent to the transcription start site (TSS) of the YTHDF1 gene. **n** Mutant HBS sequences in the YTHDF1 promoter. **o**, **p** Luciferase reporter assays for the mutant HBS sequences in SMMC7721 and Hep3B cells following HIF-1α overexpression under hypoxia. **q**, **r** Luciferase reporter assays for the mutant HBS sequences in SMMC7721 and Hep3B cells following HIF-1α knockdown under hypoxia. **s**–**t** Luciferase reporter assays for the mutant HBS sequences in SMMC7721 and Hep3B cells following LW6 treatment under hypoxia. **u**, **v** ChIP assays in SMMC7721 and Hep3B cells to investigate the binding of HIF-1α to the YTHDF1 promoter via HBS1 and HBS3 under hypoxia. Error bars represent the mean ± SEM and the dots represent the value of each experiment; *P < 0.05, **P < 0.01, ***P < 0.001, ns, no significance

HCC data mining of GEPIA database and TCGA database demonstrated a positive correlation between expression of HIF-1α and YTHDF1 (Fig. 2a, b), providing evidence for hypoxia-mediated YTHDF1 upregulation. Then, qRT-PCR and western blotting were employed to investigate the effect of HIF-1α on YTHDF1 expression under both normoxia and hypoxia. The results revealed that YTHDF1 levels were increased in HIF-1α-overexpressing HCC cells (Fig. 2c, d) and decreased in YTHDF1-knockdown HCC cells (Fig. 2e, f) under hypoxic conditions. Consistently, expression of YTHDF1 was decreased in HCC cells under hypoxia after administration of LW6,^21^ which is a novel inhibitor that selectively targets HIF-1α (Fig. 2g, h). However, similar YTHDF1 levels were obtained with and without LW6 treatment in CMV-driven FLAG-YTHDF1-overexpressing HCC cells under hypoxia, indicating that HIF-1α-mediated YTHDF1 induction had no effect on the CMV promoter, but did affect expression driven by the endogenous promoter (Fig. 2i, j). These results supported the notion that HIF-1α-mediated YTHDF1 induction occurred at the transcriptional level.

Luciferase reporter assays were then employed under both normoxia and hypoxia, demonstrating that HIF-1α binds to the YTHDF1 promoter and directly activates its transcription in SMMC7721 and Hep3B cells under hypoxia (Fig. 2k). Chromatin immunoprecipitation (ChIP) assays confirmed HIF-1α occupancy at YTHDF1 promoters in SMMC7721 and Hep3B cells under hypoxia (Fig. 2l). The online tool Jaspar was employed to identify putative HIF-1α-binding sites (HBS) in the genomic sequence adjacent to the transcription start site of the YTHDF1 gene. We observed three putative HBS within the genomic region (NM_017798) (Fig. 2m). Therefore, we mutated each HBS in turn and measured luciferase activity (Fig. 2n). When HBS1 and HBS3 were simultaneously mutated, the induction of luciferase activity by HIF-1α was completely abolished under hypoxia (Fig. 2o, p). Similarly, HIF-1α knockdown or LW6 treatment significantly reduced luciferase activity in hypoxic SMMC7721 and Hep3B cells, and mutation of HBS1 and HBS3 restored the decreased luciferase activity (Fig. 2q–t), implying that these two sites are critical for the activation of YTHDF1 transcription by HIF-1α under hypoxia. Accordingly, ChIP assays of SMMC7721 and Hep3B cells demonstrated that the chromatin fragments containing HBS1 or HBS3 were enriched with the anti-HIF-1α antibody compared to the normal IgG, while the negative control chromatin fragment did not exhibit antibody enrichment under hypoxia (Fig. 2u, v). This indicated that HIF-1α specifically bound to the YTHDF1 promoter via HBS1 and HBS3 under hypoxia. Taken together, these results identified YTHDF1 as a novel direct target gene of the HIF-1α transcription factor.

### HIF-1α/YTHDF1 signaling promotes hypoxia-induced autophagy in HCC

**Fig. 3:**
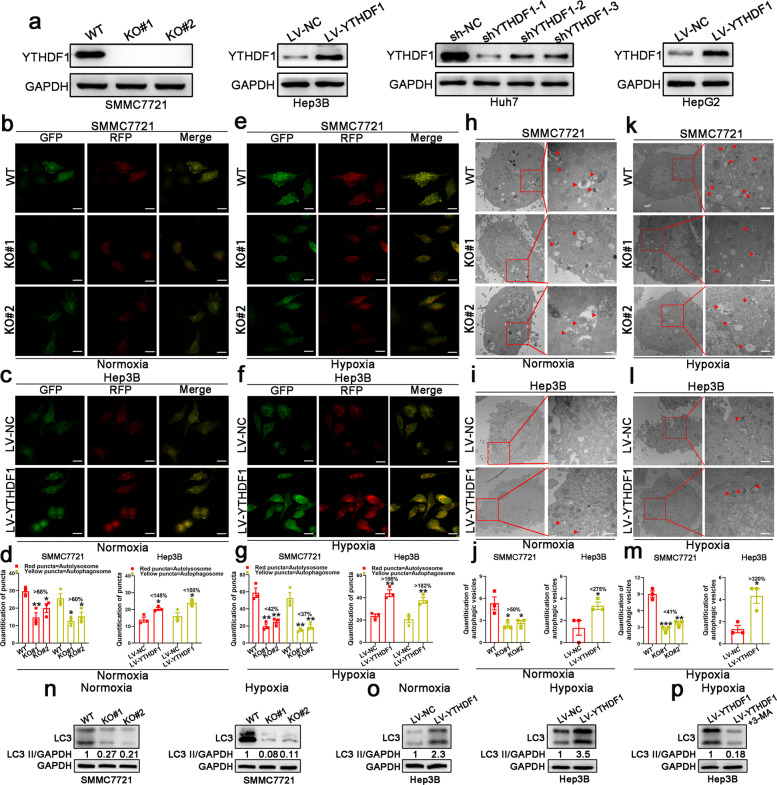
YTHDF1 promotes hypoxia-induced autophagy in HCC cell lines. **a** Protein levels of YTHDF1 in HCC cell lines determined by western blotting. **b**, **c** Immunofluorescence (IF) staining with mRFP-GFP-LC3 in normoxic SMMC7721 and Hep3B cells with YTHDF1 knockout or overexpression, respectively. Red puncta signify autolysosomes and yellow puncta signify autophagosomes. Scale bar, 10 µm. **d** Quantification of LC3 puncta under normoxia. **e**, **f** IF staining with mRFP-GFP-LC3 in hypoxic SMMC7721 and Hep3B cells with YTHDF1 knockout or overexpression, respectively. Red puncta signify autolysosomes and yellow puncta signify autophagosomes. Scale bar, 10 µm. **g** Quantification of LC3 puncta under hypoxia. **h**, **i** Transmission electron microscopy (TEM) demonstrating autolysosomes and autophagosomes in normoxic SMMC7721 and Hep3B cells with YTHDF1 knockout or overexpression, respectively. Scale bar, 1 µm. **j** Quantification of autophagic vesicles under normoxia. **k**, **l** TEM demonstrating autolysosomes and autophagosomes in hypoxic SMMC7721 and Hep3B cells with YTHDF1 knockout or overexpression, respectively. Scale bar, 1 µm. **m** Quantification of autophagic vesicles under hypoxia. **n** Western blotting demonstrating expression of LC3 in SMMC7721 cells with YTHDF1 knockout under normoxia and hypoxia. **o** Western blotting demonstrating expression of LC3 in Hep3B cells with YTHDF1 overexpression under normoxia and hypoxia. **p** Western blotting demonstrating expression of LC3 in hypoxic YTHDF1-overexpressing Hep3B cells following 3-MA treatment. Error bars represent the mean ± SEM and the dots represent the value of each experiment; *P < 0.05, **P < 0.01, ***P < 0.001

HIF-1α overexpression was shown to significantly promote autophagy in SMMC7721 cells under hypoxia, whereas inhibition of HIF-1α expression inhibited autophagy in Hep3B cells under hypoxia, as indicated by immunofluorescence (IF) staining analysis and western blotting (Supplementary Fig. S3a–f). Protein levels of YTHDF1 were determined in YTHDF1-knockout (KO) SMMC7721 cells, YTHDF1-knockdown Huh7 cells, and YTHDF1-overexpressing Hep3B and HepG2 cells (Fig. 3a). Hypoxia-induced autophagy was impaired in HIF-1α + KO#1 SMMC7721 cells compared with HIF-1α + WT SMMC7721 cells, while it was enhanced in sh-HIF-1α + LV-YTHDF1 Hep3B cells compared to sh-HIF-1α + LV-NC Hep3B cells (Supplementary Fig. S3a–f). Based on these results, we speculated that YTHDF1 was an effector of HIF-1α in hypoxia-induced autophagy of HCC.

### YTHDF1 drives hypoxia-induced autophagy in HCC

We detected autophagy reflux in HCC cells under both normoxic and hypoxic conditions. Knockout or knockdown of YTHDF1 resulted in a remarkable decrease in mRFP-GFP-LC3 dot accumulation, while overexpression of YTHDF1 had opposite effects under both normoxia and hypoxia (Fig. 3b–g and Supplementary Fig. S4a–f). Transition electron microscopy (TEM) revealed that knockout or knockdown of YTHDF1 decreased the formation of double-membrane vesicles containing cytoplasmic components, which is a characteristic of autophagy, whereas the opposite results were observed following YTHDF1 overexpression under both normoxia and hypoxia (Fig. 3h–m and Supplementary Fig. S4g–l). Western blot analysis revealed that knockout or knockdown of YTHDF1 decreased LC3 expression in SMMC7721 and Huh7 cells under both normoxia and hypoxia (Fig. 3n and Supplementary Fig. S4m). Nevertheless, increased LC3 expression was observed following YTHDF1 overexpression in Hep3B and HepG2 cells (Fig. 3o and Supplementary Fig. S4n). However, autophagy was clearly activated and affected to a greater extent by changes in YTHDF1 expression under hypoxia (Fig. 3d, g, j, m-o and Supplementary Fig. S4c, f, i, l–n), which implied that YTHDF1 played a more important role in promoting HCC autophagy under hypoxia than under normoxia. Moreover, considering that hypoxia upregulated YTHDF1 expression in a HIF-1α-dependent manner, we focused on the role of YTHDF1 in HCC cells under hypoxic conditions, which is more similar to the microenvironment in solid tumors.

### YTHDF1 promotes autophagy-associated HCC malignancy under hypoxia

Since YTHDF1 plays a more important role in promoting HCC autophagy under hypoxia, and autophagy is reportedly associated with tumor malignancy,^22^ we further investigated the tumor-promoting role of YTHDF1 and its relationship with autophagy. Knockout or knockdown of YTHDF1 in SMMC7721 and Huh7 cells significantly inhibited cell proliferation compared with controls. Conversely, YTHDF1-overexpressing Hep3B and HepG2 cells displayed a higher proliferation rate than controls (Supplementary Figs. S5a–d and S6a–d). YTHDF1 expression in HCC tissue organoids further validated the in vitro functional results. YTHDF1 knockdown and overexpression resulted in significantly decreased and increased HCC tissue organoid diameter compared with control organoids, respectively (Supplementary Fig. S5e–h).

Moreover, YTHDF1-knockout or -knockdown SMMC7721 and Huh7 cells displayed higher apoptotic rates, whereas hypoxic YTHDF1-overexpressing Hep3B and HepG2 cells exhibited contrasting results (Supplementary Figs. S5i–j and S6e–f). Cell migration and invasion were impaired following knockout or knockdown of YTHDF1 in hypoxic SMMC7721 and Huh7 cells, while they were strongly enhanced following YTHDF1 overexpression in hypoxic Hep3B cells and HepG2 cells (Supplementary Figs. S5k–l and S6g–h).

To further determine whether YTHDF1 promoted HCC malignancy under hypoxia by promoting autophagy, YTHDF1-overexpressing cells were treated with the autophagy inhibitor 3-methyladenine (3-MA, a PI3K and PtdIns3K inhibitor) (Fig. 3p and Supplementary Fig. S4o). 3-MA treatment reversed the enhanced cell proliferation, migration, and invasion of YTHDF1-overexpressing cells under hypoxia (Supplementary Figs. S5a, b, d, j, and l and S6a, b, d, f, and h), indicating that YTHDF1 could promote malignancy of HCC cells under hypoxia via autophagy activation.

### YTHDF1 deficiency inhibits HCC autophagy, growth, and metastasis in vivo

**Fig. 4:**
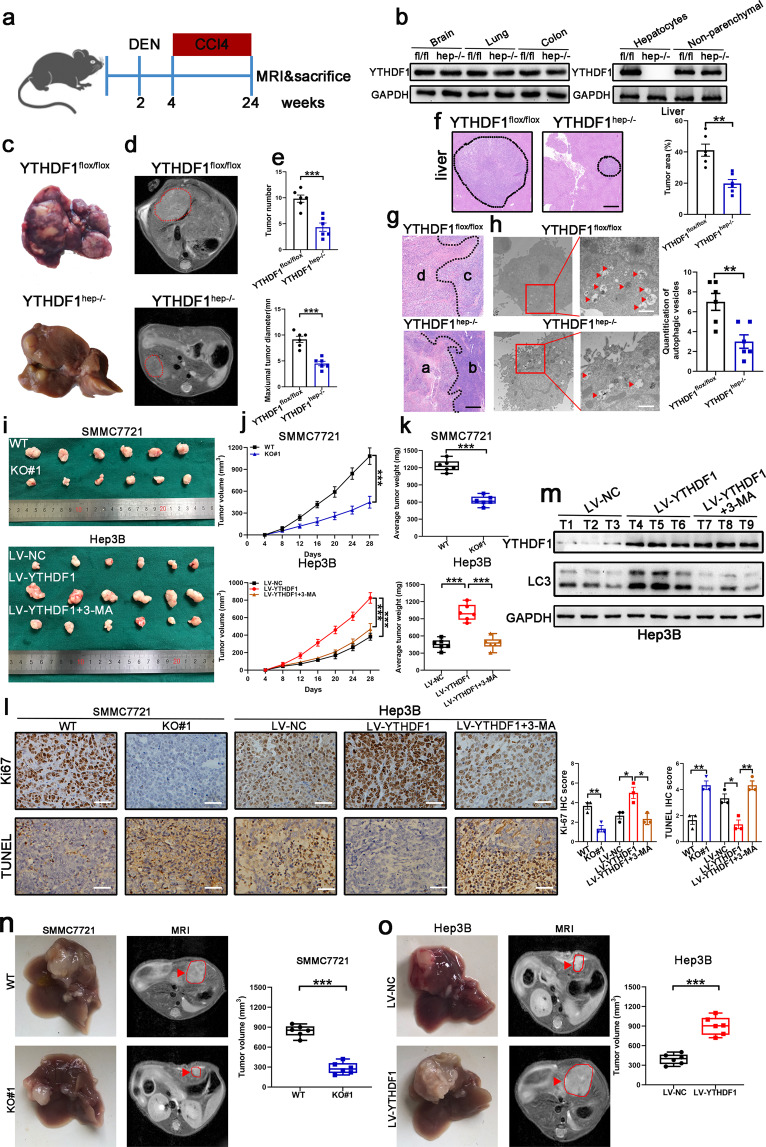
YTHDF1 deficiency inhibits HCC growth. **a** Schematic representation of the DEN/CCl4-induced HCC model. **b** YTHDF1 expression in hepatocytes and nonparenchymal cells of YTHDF1^hep−/−^ and YTHDF1^flox/flox^ mice by western blotting. **c**–**e** Representative gross appearance (**c**), magnetic resonance imaging (MRI) (**d**), tumor number, and maximum tumor diameter (**e**) of livers from DEN/CCl4-treated YTHDF1^hep−/−^ and YTHDF1^flox/flox^ mice. **f** Hematoxylin and eosin (H&E) staining of liver sections from DEN/CCl4-treated YTHDF1^hep−/−^ and YTHDF1^flox/flox^ mice. Scale bar, 200 µm. **g** H&E staining for representative tumor sections. Scale bar, 200 µm. **h** TEM showing the ultra-microstructure of the tumor section. Red arrows indicate autophagosomes or autolysosomes that have a double-layer structure. Scale bar, 1 µm. **i** Representative xenograft tumors after subcutaneous injection of SMMC7721 cells transfected with YTHDF1-KO#1 and WT 28 d after inoculation (upper). Representative xenograft tumors after subcutaneous injection of Hep3B cells transfected with LV-YTHDF1 and corresponding control 28 d after inoculation (low). 3-MA was used as an autophagy inhibitor. **j** Time course of HCC xenograft growth. **k** Tumor weight of HCC xenografts. **l** Proliferation (Ki67) and apoptosis (Tunel) immunohistochemistry (IHC) staining of tumor sections. Scale bar, 100 µm. **m** Western blotting of YTHDF1 and LC3 proteins. 3-MA was used as an autophagy inhibitor. **n** Nude mice orthotopically implanted tumors of YTHDF1-knockout SMMC7721 cells. **o** Nude mice orthotopically implanted tumors of YTHDF1-overexpressing Hep3B cells. Error bars represent the mean ± SEM and the dots represent the value of each experiment; *P < 0.05, **P < 0.01, ***P < 0.001

Besides the tumor-promoting effects of YTHDF1 in vitro, we also investigated this effect in YTHDF1^flox/flox^ mice using a conditional knockout strategy to edit the YTHDF1 gene, as shown in Supplementary Fig. S7a, and an *N*-nitrosodiethylamine (DEN)-induced HCC model (Fig. 4a). YTHDF1 protein expression was unaffected in other tissues, and its expression in the liver was specifically ablated in hepatocytes but was present in the nonparenchymal cells of YTHDF1^hep−/−^ mice (Fig. 4b). Compared to YTHDF1^flox/flox^ littermates, YTHDF1^hep−/−^ mice developed less advanced liver lesions, assessed by gross appearance (Fig. 4c), magnetic resonance imaging (MRI) (Fig. 4d), tumor number, maximum tumor diameter (Fig. 4e), and histology (Fig. 4f). A transitional zone, characterized by irregular, large hyperchromatic nuclei between the necrotic area (Fig. 4g, areas a) and tumor cells with regular morphology (Fig. 4g, areas b), was either not present or only present in a narrow area in the tumors of YTHDF1^hep−/−^ mice. The results demonstrated that the tumor cells of YTHDF1^hep−/−^ mice were prone to necrosis due to difficulty adapting to survival stress such as hypoxia in vivo. However, a distinct transitional zone (Fig. 4g, area c) between the necrotic area (Fig. 4g, area d) and tumor cells with regular morphology was observed in the tumors of YTHDF1^flox/flox^ mice. This indicates that the tumor cells tried to adjust to the stress but finally died upon decompensation. Additionally, TEM results demonstrated that tumor cells of YTHDF1^hep−/−^ mice had fewer autophagic vacuoles adjacent to the necrotic area. However, the tumor cells of YTHDF1^flox/flox^ mice demonstrated high autophagic activity, which was indicative of their efforts to adapt to stress before death occurred (Fig. 4h).

To further validate whether YTHDF1 promoted autophagy and tumorigenesis in vivo, we subcutaneously injected HCC cells into nude mice. The growth of YTHDF1-KO#1 transfected subcutaneous tumors was hindered compared to that of the controls (SMMC7721 cells), while that of LV-YTHDF1-transfected subcutaneous tumors was faster than that of the controls (Hep3B cells) (Fig. 4i–k). Further, significantly decreased proliferation and increased apoptosis was observed for the YTHDF1-KO#1 transfected subcutaneous tumors. However, opposite results were observed for LV-YTHDF1-transfected subcutaneous tumors (Fig. 4l). Western blot analysis demonstrated increased YTHDF1 protein levels accompanied by increased LC3 protein levels in LV-YTHDF1-transfected tumors (Fig. 4m).

Next, we analyzed the effect of autophagy following YTHDF1 overexpression in vivo by employing intraperitoneal injections of 3-MA at regular intervals to inhibit autophagy (Fig. 4m). 3-MA treatment reduced the increased tumor formation of Hep3B cells with YTHDF1 overexpression (Fig. 4i–k, lower). IHC staining of LV-YTHDF1-transfected subcutaneous tumors also demonstrated that increased Ki67 scoring and decreased TUNEL scoring were recovered by 3-MA treatment (Fig. 4l). The biological effect of YTHDF1 was further elucidated in vivo by orthotopic tumor transplantation. Tumor growth was remarkably suppressed in the YTHDF1-KO#1 group, while the opposite phenomenon was observed in the LV-YTHDF1 group (Fig. 4n–o).

The effect of YTHDF1 on lung metastasis was evaluated in vivo. Bioluminescence imaging results demonstrated that YTHDF1 knockout markedly inhibited lung metastasis in this model (Supplementary Fig. S8a), which was further validated by H&E staining of lung tissues (Supplementary Fig. S8b, c). In contrast, incidence of lung metastases was increased in the YTHDF1 overexpression group (Supplementary Fig. S8a–c). IHC staining demonstrated that lung metastases incidence was positively associated with YTHDF1 level in the metastases (Supplementary Fig. S8d). YTHDF1-knockout and -overexpressing mice exhibited longer and shorter OS, respectively, than control mice (Supplementary Fig. S8e). Taken together, these results demonstrated that YTHDF1 deficiency inhibited HCC autophagy, growth, and metastasis.

### The oncogenic function of YTHDF1 is dependent on m6A-binding pockets in the YTH domain

YTHDF1 is known to bind m6A sites via its m6A-binding pockets in the YTH domain. Mutations in K395 and Y397 could abrogate the binding capacity of YTHDF1 with mRNA (Supplementary Fig. S9a). Therefore, we evaluated whether m6A-binding pockets in the YTH domain of YTHDF1 were essential for oncogenic activity in HCC hypoxia-induced autophagy and malignancy. SMMC7721 and Hep3B cells were both individually transfected with wild-type YTHDF1 (YTHDF1-WT) or YTHDF1-MUT (with K395A and Y397A two point mutations in the YTH domain of YTHDF1 with FLAG tag) constructs. Autophagy was significantly increased in YTHDF1-WT but not YTHDF1-MUT SMMC7721 cells under hypoxia (Supplementary Fig. S9b, c). Moreover, overexpression of YTHDF1-WT but not YTHDF1-MUT was shown to efficiently promote HCC cell proliferation, migration, and invasion under hypoxia (Supplementary Fig. S9d–g). A comparable phenomenon was also observed in Hep3B cells using the same strategy (Supplementary Fig. S9h–m). These results indicated that the m6A-binding pockets in the YTH domain of YTHDF1 were indispensable for the regulation of hypoxia-induced autophagy and malignancy.

### Translation of ATG2A and ATG14 is regulated by YTHDF1

**Fig. 5:**
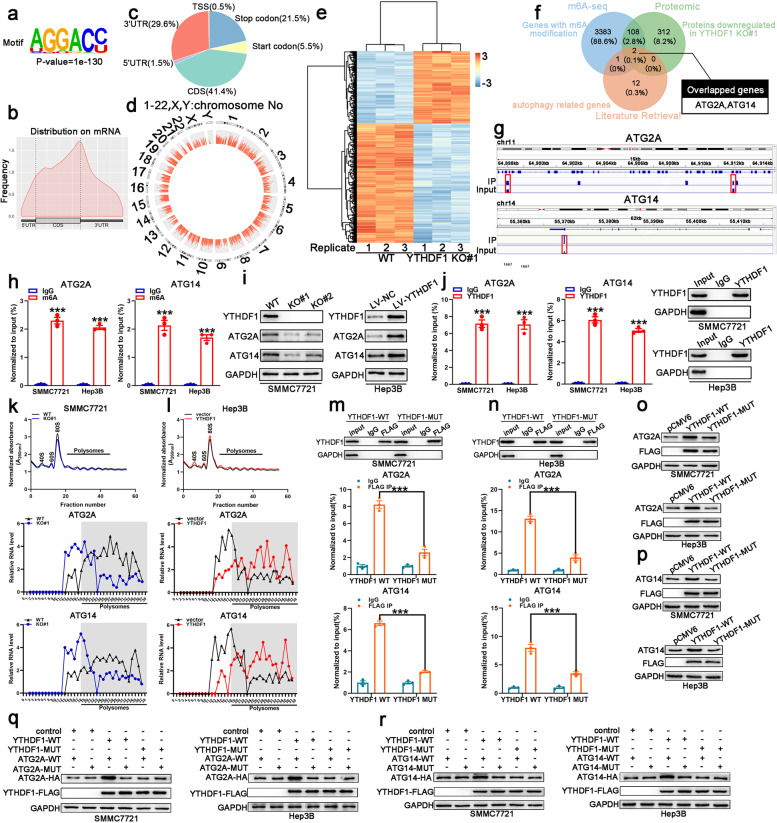
MeRIP-seq and proteomics identified potential targets of YTHDF1 in HCC. **a** m6A motif detected by the MEME motif analysis with MeRIP-seq data in hypoxic SMMC7721 cells. **b** Metagene profiles of m6A enrichment across mRNA transcriptome in hypoxic SMMC7721 cells. **c** Distribution of m6A sites within different gene regions. **d** Circos plot displaying the distribution of m6A peaks in the human transcriptome of hypoxic SMMC7721 cells. **e** Heat map showing the most significantly altered proteins. **f** Overlapping analysis of genes identified by MeRIP-seq, proteomics, and autophagy-related genes. **g** Integrative genomics viewer (IGV) plots of m6A peaks at ATG2A and ATG14 mRNAs. **h** Methylated RNA immunoprecipitation of the transcripts of ATG2A and ATG14 in hypoxic SMMC7721 and Hep3B cells. **i** Validation of ATG2A and ATG14 expression in hypoxic SMMC7721 and Hep3B cells with YTHDF1 knockout or overexpression, respectively. **j** YTHDF1 immunoprecipitation assays of ATG2A and ATG14 transcripts in YTHDF1-bound mRNAs in hypoxic SMMC7721 and Hep3B cells. **k**, **l** Polysome profiling of hypoxic SMMC7721 and Hep3B cells with YTHDF1 knockout (**l**) or overexpression (**l**), respectively (upper panel); qRT-PCR analysis of ATG2A (middle panel) and ATG14 (lower panel) mRNA distribution in different ribosome populations. **m** RIP-derived protein and RNA in hypoxic SMMC7721 cells examined using western blotting and RT-qPCR, respectively. GAPDH was employed as a negative control in western blotting. **n** RIP-derived protein and RNA in hypoxic Hep3B cells examined using western blotting and RT-qPCR, respectively. GAPDH was employed as a negative control in western blotting. **o**, **p** Analysis of ATG2A and ATG14 expression following overexpression of YTHDF1 wild-type or mutant using western blotting. **q**, **r** Western blotting detected HA-tagged ATG2A and ATG14 expression in hypoxic SMMC7721 and Hep3B cells co-transfected with empty vector, wild-type, or mutant FLAG-tagged YTHDF1, and wild-type or mutant HA-tagged ATG14 or ATG2A. Error bars represent the mean ± SEM and the dots represent the value of each experiment; ***P < 0.001

YTHDF1 functions as an m6A reader, affecting ribosome occupancy and translation of m6A-modified mRNAs.^23^ To identify potential mRNA targets of YTHDF1, hypoxic SMMC7721 cells were selected for methylated RNA immunoprecipitation sequencing (MeRIP-seq) and proteomics analysis. MeRIP-seq identified 4918 m6A peaks in 3494 genes (Supplementary Table S4). Multiple Expression motifs for Motif Elicitation (MEME) algorithm analysis identified the m6A consensus motif (GGAC), indicating the successful enrichment of m6A-modifed mRNA (Fig. 5a). In line with other m6A-seq results, these m6A modifications were preferentially enriched in coding regions (CDS) and 3′-untranslated regions (3′-UTR) (Fig. 5b, c). Figure 5d illustrates a circos plot representing the distribution of methylated m6A peaks in the transcriptome of human hypoxic SMMC7721 cells. Meanwhile, a total of 678 proteins were significantly changed in YTHDF1 knockout cells (fold-change ≥ 1.5, *P* < 0.05) (Fig. 5e and Supplementary Table S5). ATG2A and ATG14 were determined as the potential target genes of YTHDF1 by overlapping the genes identified by MeRIP-seq, proteomics, and autophagy-associated genes (ATG101, ATG2A, ATG12, ATG7, ATG16L1, ATG9A, ATG2B, ATG10, ATG5, ATG3, ATG4B, ULK1, ATG4D, ATG13, ATG14) (Fig. 5f). Integrative genomics viewer (IGV) plots of m6A peaks at ATG2A and ATG14 mRNAs are shown in Fig. 5g. The m6A modification status of ATG2A and ATG14 mRNA was assessed by the gene-specific m6A assay. ATG2A and ATG14 mRNAs were significantly enriched in both hypoxic SMMC7721 and Hep3B cells (Fig. 5h). Western blot analysis demonstrated that ATG2A and ATG14 expression decreased in YTHDF1-knockout cells, while increasing in YTHDF1-overexpressing cells (Fig. 5i). Moreover, the interaction between YTHDF1 and ATG2A or ATG14 mRNA in both hypoxic SMMC7721 and Hep3B cells was confirmed using the YTHDF1-specific antibody in RIP-qPCR analysis (Fig. 5j).

Treatment of HCC cells with actinomycin D (an inhibitor of transcription) revealed that knockout or overexpression of YTHDF1 did not affect the stability of ATG2A and ATG14 mRNA in both hypoxic SMMC7721 and Hep3B cells, as reflected in the comparable half-lives (*t*_1/2_) of ATG2A and ATG14 mRNA following YTHDF1 knockout or overexpression (Supplementary Fig. S10a–d). Since ATG2A and ATG14 protein levels declined under YTHDF1-deficient conditions, we also treated SMMC7721 and Hep3B cells with the protein translation inhibitor cycloheximide (CHX) to exclude the possibility that YTHDF1 contributed to ATG2A and ATG14 protein stability. YTHDF1 knockout or overexpression did not affect the stability of ATG2A and ATG14 in both hypoxic SMMC7721 and Hep3B cells (Supplementary Fig. S10e–h). However, polysome profiling demonstrated that YTHDF1 knockout resulted in a moderate shift of ATG2A and ATG14 mRNAs to non-polysome fractions with decreased ATG2A and ATG14 mRNAs in the translation fractions (Fig. 5k). Conversely, overexpression of YTHDF1 resulted in a shift of ATG2A and ATG14 mRNAs to polysome fractions with increased ATG2A and ATG14 mRNAs in the translation fractions, indicating that YTHDF1 regulated protein synthesis of ATG2A and ATG14 (Fig. 5l).

Subsequently, RIP analysis demonstrated that ATG2A or ATG14 mRNA was effectively immunoprecipitated in SMMC7721 and Hep3B cells transfected with YTHDF1-WT, but the interaction between YTHDF1-MUT and ATG2A or ATG14 mRNA was significantly decreased, indicating that the m6A-binding pocket of YTHDF1 was crucial for its binding to ATG2A and ATG14 mRNA (Fig. 5m, n). Moreover, we observed that YTHDF1-WT, but not YTHDF1-MUT, upregulated protein expression of ATG2A and ATG14 (Fig. 5o, p). Additionally, HA-tagged ATG2A and ATG14 expression vectors (ATG2A-WT/ATG14-WT) and their mutant with m6A site mutations (ATG2A-MUT/ATG14-MUT) were constructed (Supplementary Fig. S10i, j). Western blot analysis demonstrated that YTHDF1-WT but not YTHDF1-MUT could increase the expression of ATG2A-WT and ATG14-WT, while YTHDF1-WT did not affect the expression of ATG2A-MUT and ATG14-MUT (Fig. 5q, r).

### Ectopic expression of ATG2A or ATG14 compensates for the tumor-suppressive effect of YTHDF1 deficiency in HCC

Rescue experiments were performed to explore whether ATG2A and ATG14 participated in the oncogenic function of YTHDF1 in HCC. SMMC7721-WT and YTHDF1-knockout cells were transfected with control, ATG2A, or ATG14 overexpression lentivirus. Ectopic expression of ATG2A and ATG14 in YTHDF1-knockout SMMC7721 cells partially abolished inhibition of hypoxia-induced autophagy (Supplementary Fig. S11a–e). Ectopic expression of ATG2A and ATG14 in YTHDF1-knockout SMMC7721 cells also partially abolished inhibition of HCC cell malignancy under hypoxia (Supplementary Fig. S12a, b, e, g). Meanwhile, downregulation of ATG2A or ATG14 in YTHDF1-overexpressing Hep3B cells treated with ATG2A or ATG14 shRNA significantly corrected the YTHDF1-mediated increase in hypoxia-induced autophagy and malignancy (Supplementary Figs. S11f–j, S12c, d, f, and h).

Xenografts in nude mice were generated to further determine whether ATG2A or ATG14 is a critical downstream target of YTHDF1 that facilitates HCC hypoxia-induced autophagy and malignancy. Decreased volume and weight of the xenograft tumors in the YTHDF1-knockout group was rescued by ectopic expression of ATG2A or ATG14 in SMMC7721 cells (Supplementary Fig. S13a–c). Similarly, ATG2A or ATG14 downregulation in Hep3B cells counteracted the stimulative effect of YTHDF1 overexpression on tumor growth (Supplementary Fig. S13d–f). In metastatic models, inhibition of lung metastasis by YTHDF1 knockout was more pronounced in SMMC7721 cells with ATG2A or ATG14 overexpression (Supplementary Fig. S13g). Histological analysis of lung tissues confirmed the aforementioned effects (Supplementary Fig. S13i, k). In accordance with this, ATG2A or ATG14 downregulation in Hep3B cells robustly reversed the promotive effect of YTHDF1 overexpression on lung metastasis, as validated using in vivo imaging and histological analysis (Supplementary Fig. S13h, j, and l). Collectively, these results indicated that ATG2A and ATG14 are the downstream targets of YTHDF1 and mediate the role of YTHDF1 in HCC progression.

### YTHDF1 facilitates HCC growth in a patient-derived xenograft (PDX) mouse model

**Fig. 6:**
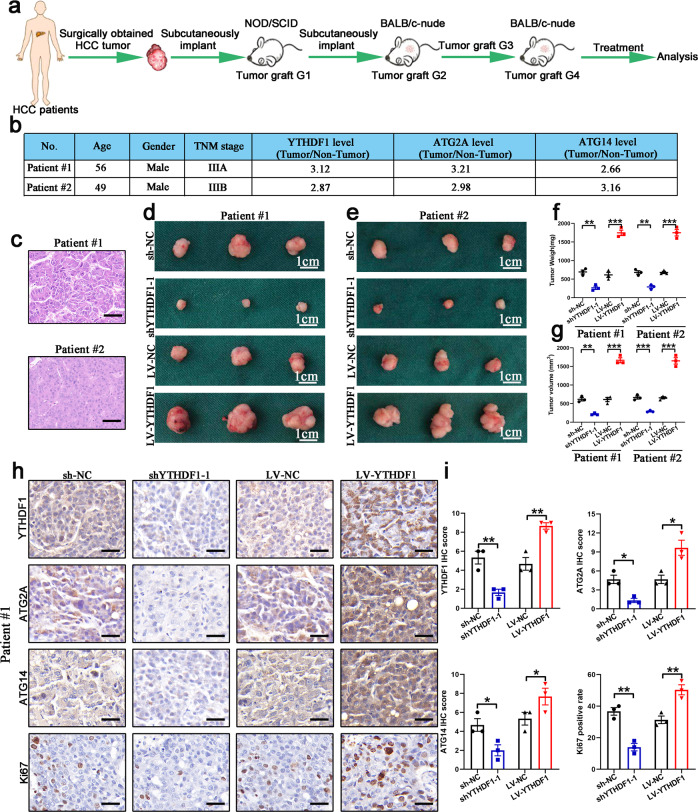
YTHDF1 deficiency inhibits HCC growth in PDX models. **a** Graphic illustration of HCC PDX mouse models. **b** Clinical characteristics of the donor patients. **c** Hematoxylin and eosin (H&E) staining of donor patient tissues. Scale bar, 100 µm. **d**, **e** Harvested engrafted tumors in the sh-NC, shYTHDF1, LV-NC, LV-YTHDF1 groups. **f** Tumor weight of the engrafted tumors. **g** Tumor volume of the engrafted tumors. **h**, **i** Expression levels of YTHDF1, ATG2A, ATG14, and Ki67 in PDX tumor tissues determined by immunohistochemistry (IHC) staining. Scale bar, 50 µm. Error bars represent the mean ± SEM and the dots represent the value of each experiment; *P < 0.05, **P < 0.01, ***P < 0.001

To further validate that changes in YTHDF1 expression could therapeutically benefit HCC patients, we generated two HCC PDX murine models (Fig. 6a). The clinical characteristics of the donor patients are shown in Fig. 6b. Histopathological analysis of the engrafted tumors (Fig. 6c) revealed that YTHDF1 knockdown induced a remarkable decrease in tumor growth, while YTHDF1 overexpression promoted tumor growth (Fig. 6d–g). IHC staining confirmed decreased YTHDF1 expression in the sh-YTHDF1-1 group and increased YTHDF1 expression in the LV-YTHDF1 group (Fig. 6h, i). IHC results further demonstrated downregulated ATG2A, ATG14, and Ki67 expression levels in the sh-YTHDF1-1 group and upregulated ATG2A, ATG14, and Ki67 expression levels in the LV-YTHDF1 group (Fig. 6h, i).

### Levels of YTHDF1 and ATG2A or ATG14 are clinically relevant in patients with HCC

Positive correlations were determined between expression of YTHDF1 and ATG2A (*R* = 0.550; *P* < 0.001), and between YTHDF1 and ATG14 (*R* = 0.538; *P* < 0.001) (Supplementary Fig. S14a, b). Patients with high ATG2A or ATG14 expression experienced shorter DFS and OS than patients with low ATG2A or ATG14 expression. The shortest DFS and OS were detected in patients with three highly expressed markers, YTHDF1/ATG2A/ATG14 (Supplementary Fig. S14c–h). These results indicated that elevated expression levels of YTHDF1 and its target genes ATG2A and ATG14 may identify HCC patients with poor prognosis.

## Discussion

Accumulating evidence indicates that m6A methylation dysregulation is closely associated with the development and progression of various malignancies.^24^ Further, m6A methyltransferases or demethylases either facilitate or impair carcinogenesis in different malignancies. For example, the m6A methyltransferase METTL14 inhibits tumorigenesis in colorectal cancer, bladder cancer, and HCC.^19,25,26^ However, METTL14 also plays an oncogenic role by regulating the expression of its mRNA targets (e.g., MYB and MYC) via m6A modification in leukemogenesis.^27^ Owing to recognition by diverse m6A readers, mRNA transcripts with m6A modifications are subjected to different fates. YTHDC2, YTHDF2, and YTHDF3 promote the decay of their target mRNAs, thereby reducing the expression of their target genes; IGF2BPs promote mRNA stability; while YTHDF1 promotes the translation of targeted mRNA, thus promoting gene expression.^23,28^

Hypoxia acts as a driver in the development and progression of neoplasms.^29^ Hypoxia-induced HIF stabilization correlates with poor survival of cancer patients.^30^ HIF-1α is an extensively investigated HIF that responds to hypoxic stress by activating transcription of downstream target genes. HIF-1α undergoes proteasome degradation under normoxic conditions. However, hypoxic stress protects HIF-1α from proteasome degradation, resulting in HIF-1α translocation into the nucleus and transcription initiation.^31^ Here, we observed that hypoxia upregulated YTHDF1 expression in an HIF-1α-dependent manner by specifically binding to the YTHDF1 promoter via both HBS1 and HBS3. Moreover, increased YTHDF1 expression correlated with poor prognosis in patients with HCC. Including YTHDF1 expression in clinical risk scores could enhance predictive ability, indicating that YTHDF1 may be a biomarker for HCC.

Previous studies have highlighted that autophagy is essential for cancer cell survival under stress. Chang et al.^9^ demonstrated that activation of autophagy served as a protective response under hypoxia in HCC. Lin et al.^32^ demonstrated that hypoxia-induced autophagy led to chemoresistance in HCC cells. Additionally, Gao et al.^33^ reported that circCDR1as activated autophagy under hypoxia in other malignancies, such as oral squamous cell carcinoma, contributing to cancer cell survival. However, the biological function of m6A modification in hypoxia-induced autophagy remained unknown. Herein, we demonstrated that YTHDF1 contributed to hypoxia-induced autophagy and autophagy-related malignancy of HCC in multiple in vitro and in vivo HCC models. These results concurred with the reported oncogenic role of YTHDF1 in gastric cancer, ovarian cancer, Merkel cell carcinoma, melanoma, and non-small cell lung cancer.^28,34–37^ PDX models have been successfully utilized to investigate human malignancies for preclinical therapeutics.^38,39^ Our experiments with HCC PDX indicated that targeting YTHDF1 might prove to be a promising strategy for HCC treatment.

To investigate the underlying mechanisms of YTHDF1 in hypoxia-induced autophagy and autophagy-related malignancy of HCC, we applied MeRIP-seq and proteomics analysis to identify potential autophagy-related genes. Our results indicated that ATG2A and ATG14 are the critical target genes of YTHDF1 in HCC, as shown in the proposed model illustrated in Supplementary Fig. S14i. As a m6A modification reader, YTHDF1 directly binds and recognizes m6A methylation of ATG2A and ATG14 mainly at the CDS, thus promoting their translation by recruiting initiation factors and facilitating ribosome loading. Moreover, our mutagenesis assays suggested that the m6A sites in the CDS of ATG2A and ATG14 are indispensable for regulating translation of ATG2A and ATG14 by YTHDF1. Previous studies have demonstrated that ATG2A and ATG14 are key drivers in promoting autophagy and malignant biological behavior in HCC.^22,40^ Here, we also demonstrated that ATG2A and ATG14 are involved in YTHDF1-mediated hypoxia-induced autophagy and autophagy-related malignancy in HCC. Higher expression levels of YTHDF1, ATG2A, and ATG14 were associated with worse outcomes in patients with HCC, indicating that YTHDF1 and its target genes ATG2A and ATG14 may serve as promising biomarkers in predicting prognosis and in developing treatment strategies for HCC.

## Conclusions

In conclusion, our study demonstrated that YTHDF1 expression promoted both hypoxia-induced autophagy and autophagy-associated malignancy of HCC by contributing to the translation of its target genes, ATG2A and ATG14. Of note, HIF-1α-mediated upregulation of YTHDF1 under hypoxia in HCC cells provides evidence that adaptation of cancer epigenetics to hypoxic stress forces HCC malignancy, at least in part, for the translation of m6A-containing oncogene mRNAs. Taken together, our study indicates that m6A modification of mRNA in HCC and YTHDF1 might provide potential candidates for HCC treatment.

## Materials and methods

### Tissue specimen collection and cell culture

This study was approved by the Institutional Ethical Board of the First Affiliated Hospital of Nanjing Medical University. Patients or their relatives provided informed consent for use of tissue samples and data. HCC and adjacent non-tumor specimens were obtained from 120 patients who underwent hepatectomy at the Hepatobiliary Center of The First Affiliated Hospital of Nanjing Medical University between February 2013 and December 2019, as previously described.^41^

HCC cell lines were purchased from the Type Culture Collection of the Chinese Academy of Sciences (Shanghai, China) and cultured as per the established protocol.^5^ We established YTHDF1-knockout SMMC7721 cells using the CRISPR-Cas9 genome editing system and generated stable YTHDF1 knockdown Huh7 cells. We also established stable YTHDF1-overexpressing HCC cells (Hep3B and HepG2).

### Cell proliferation assays

The cell counting kit-8 (CCK-8) (Dojindo Laboratories, Kumamoto, Japan) and 5-ethynyl-2′-deoxyuridine (EdU) assay kit (RiboBio, Guangzhou, China) were used to evaluate cell proliferation according to the manufacturers’ instructions.

### Colony formation assay

Five hundred cells per well were seeded into 6-well plates and cultured in an incubator for 10 days. Colonies comprising ≥50 cells were stained with 0.1% crystal violet dye (Beyotime Biotech, Guangzhou, China) and subsequently counted.

### Cell transfection and analysis of autophagic flux

Lentiviral transfection of HCC cells was performed using mRFP-GFP-LC3 (GeneChem Co., Ltd., Shanghai, China), according to the manufacturer’s instructions. Following puromycin selection for 2 weeks, images were captured by confocal fluorescence microscopy (Carl Zeiss, Oberkochen, Germany). Yellow or red fluorescence was quantified to monitor the progression of autophagic flux under normoxia or hypoxia. The number of puncta per cell was counted in five random images. TEM was performed as previously described.^11^

### Flow cytometry

Apoptotic cells were stained with 50 μg/mL Annexin V-FITC (Becton, Dickinson and Company, Franklin Lakes, NJ, USA) and 10 μg/mL PI (Sigma-Aldrich) for 10 min at 37 °C. Cells were analyzed for apoptosis using a FACScan flow cytometer (Becton, Dickinson and Company).

### RNA extraction and qRT-PCR

Total RNA was extracted from HCC cells and frozen tissues using TRIzol reagent (Invitrogen, Carlsbad, CA, USA). mRNA reverse transcription was performed using the PrimeScript RT Master Mix Kit (TaKaRa Bio Inc., Kusatsu, Japan), according to the manufacturer’s instructions. qRT-PCR was performed using the SYBR Green PCR Kit (TaKaRa Bio Inc.) with β-actin as the internal control for gene expression. The primer sequences used were: YTHDF1 forward primer-CACCCAGAGAACAAAAGGACAAG; YTHDF1 reverse primer- CGGCGGGTAATAGCTGGAC.

### Chromatin immunoprecipitation (ChIP) assay

HCC cells were cross-linked with 1% (v/v) formaldehyde in PBS for 10 min at 37 °C. Subsequently, 0.125 M glycine was added to terminate the reaction and cells were lysed on ice using lysis buffer. Chromatin DNA was sonicated to obtain ~500 bp fragments, followed by incubation with anti-HIF-1α antibody. HIF-1α-bound chromatin was precipitated with protein G-agarose. After de-crosslinking, qPCR was employed to analyze the precipitated DNA to determine putative HIF-1α binding sites in YTHDF1 promoter regions.

### Dual luciferase assay

HCC cells were seeded in 24-well plates and incubated until they reached 70% confluence. They were then transfected with 0.75 μg pGL3-basic-YTHDF1 promoter-luciferase reporter and 0.25 μg HIF-1α expression plasmid or empty vector along with 0.025 μg pRL-TK for normalization. Luciferase activity was measured after 48 h according to the manufacturer’s instructions. The activity of the pGL3-basic-YTHDF1 promoter-luciferase reporter was normalized to that of the pRL-TK Renilla luciferase reporter, and compared between HIF-1α overexpression or knockdown HCC cells and those transfected with empty vector.

### RNA immunoprecipitation

HCC cells were harvested and suspended in IP lysis buffer. After incubation on ice for 30 min, the lysate was collected after centrifugation at 12,000 × *g* for 10 min. Antibodies or pre-immune IgG were added to the lysate, followed by rotating overnight at 4 °C. After washing the beads twice, TRIzol reagent (Invitrogen) was used to extract the RNA according to the manufacturer’s protocol.

### RNA stability assay

HCC cells were treated with 5 μg/mL actinomycin D for 0, 3, 6, and 9 h. The degradation rate and half-life of ATG2A or ATG14 mRNA were estimated according to the method described by a previous study.^14^ Briefly, the degradation rate of mRNA (*K*_decay_) was calculated by the following equation:

$${\mathrm{ln}}\left( {C/C0} \right) = - K_{{\mathrm{decay}}}{t}$$

where *t* is the transcription inhibition time, and *C* is the mRNA level at time *t*. *C*0 is the level of mRNA at 0 h in the equation, i.e., before decay starts. Thus, the mRNA half-time (*t*_1/2_) can be calculated by the equation:

$${\mathrm{ln}}\left( {1/2} \right) = - K_{{\mathrm{decay}}}t_{{\mathrm{1}}/{\mathrm{2}}}.$$

### Protein stability

Cycloheximide (100 μg/mL) (CHX; Merck Millipore, Darmstadt, Germany) was added to HCC cells at predetermined intervals. Cells were harvested, and the protein stability of ATG2A and ATG14 was determined using western blotting.

### Western blotting

Western blotting was carried out as previously described,^42^ using anti-LC3 (Abcam, Cambridge, UK), anti-ATG2A (Cell Signaling Technology Inc., Danvers, MA, USA), anti-ATG14 (Cell Signaling Technology Inc.), and anti-YTHDF1 (Proteintech, Wuhan, China). Secondary antibodies were obtained from Sigma-Aldrich.

### Polysome profiling

Prior to harvesting, HCC cells were treated with CHX (100 μg/mL) for 10 min at 37 °C. Cells were collected and lysed on ice with lysis buffer. The lysate was loaded onto a 10/50% (w/v) sucrose gradient prepared in lysis buffer, and then centrifuged at 27,500 rpm for 4 h at 4 °C. The sample was fractionated and analyzed using the Gradient Station (BioComp Instruments, Fredericton, Canada). RNA was purified from each fraction and subjected to RT-qPCR analysis.

### Construction of a hepatocellular carcinoma tissue organoid model

HCC tissue organoid models were established as described in our previous study,^5^ using fresh tumor tissues from patients with HCC. Photographs of the HCC tissue organoids were taken daily. Tissue organoids were transfected with shYHTDF1-1, LV-YHTDF1, or their control lentivirus. HCC tissue organoids were harvested after incubation for 10 days in Matrigel.

### Animal studies

Induction of carcinogenesis: A liver-specific YTHDF1 KO mouse strain (YTHDF1^hep−/−^) was generated by crossing YTHDF1^flox/flox^ with Albumin-Cre transgenic mice. Male YTHDF1^hep−/−^ mice (C57BL/6 background) and their corresponding wild-type (YTHDF1^flox/flox^) littermates were obtained from the Animal Research Center of Nanjing University. Fourteen-day-old YTHDF1^hep−/−^ and YTHDF1^flox/ flox^ mice were administered 25 mg/kg DEN (Sigma-Aldrich) via intraperitoneal (i.p.) injection to induce hepatocarcinogenesis. Two weeks after DEN injection, mice were administered 0.5 µL/g carbon tetrachloride (CCl_4_) dissolved in corn oil via i.p. injection once weekly, for a total of 20 weeks. DEN/CCl_4_-treated mice were then euthanized, and their livers were resected and visually examined for gross lesions. The number of tumors was determined. Liver tumor tissues were snap-frozen in liquid nitrogen or were fixed in 4% formalin, embedded in paraffin, and sectioned for H&E staining.

Subcutaneous and orthotopic models: HCC cells (1 × 10^6^/100 μL PBS) were administered to 4-week-old female BALB/c nude mice by subcutaneous injection (*n* = 6). The length (L) and width (W) of tumors were monitored using Vernier calipers every 4 days. Tumor volume was calculated as follows: volume (mm^3^) = (*W*^2^ × *L*)/2. For orthotopic models, tumors from the aforementioned subcutaneous HCC models were minced into 1–2 mm^3^ cubes and transplanted to the livers of BALB/c mice. Four weeks after transplantation, MRI was performed, and the mice were euthanized.

Lung metastasis models: HCC cells (1 × 10^6^/100 μL PBS) transfected with lentiviral vectors expressing the luciferase gene were injected into 4-week-old female BALB/c nude mice via the tail vein. Six weeks after injection, the bioluminescent signals after injecting 100 mg/kg D-luciferin (Xenogen, Hopkinton, MA) in mice were captured using an IVIS 100 Imaging System (Xenogen). The lungs were excised and fixed in 4% paraformaldehyde for H&E staining. The presence of lung metastatic foci was confirmed, and foci were counted microscopically by routine histopathological analysis. The remaining mice were monitored for survival analysis with 12 weeks as a cutoff.

### Patient-derived xenograft model (PDX model)

NOD/SCID and BALB/c mice were used to establish the HCC PDX model. Briefly, tumor tissues were collected from two patients with HCC after surgical resection at the Hepatobiliary Center, The First Affiliated Hospital of Nanjing Medical University, and stored on iced Dulbecco’s modified Eagle’s medium (DMEM; Gibco, Carlsbad, CA, USA) supplemented with 10% fetal bovine serum and antibiotics. The tumor tissues were then cut into 2–3 mm^3^ pieces and subcutaneously implanted into the flanks of NOD/SCID mice. The tissues from mice bearing PDX tumors were harvested and cut into pieces when the tumor volume reached 1–2 cm^3^, and then implanted into BALB/c nude mice. When the xenografted tumors grew to 50 mm^3^, recombinant lentivirus vectors were intratumorally injected into the tumor tissues for 20 days. Tumor weight and volume were recorded. The tumors were harvested for further analysis.

### Immunohistochemical staining (IHC)

HCC tissues and xenografts used for IHC were prepared as previously described.^43^ The positive IHC rate and IHC intensity scores were multiplied to calculate the overall score.

### Statistical analysis

Data are presented as the mean ± standard error of the mean (SEM). Statistical analysis was performed using IBM SPSS Statistics v20.0 (IBM Corp., Armonk, NY, USA) or GraphPad Prism v8.01 (GraphPad Software, La Jolla, CA, USA). The association between clinical characteristics of the patients and expression of YTHDF1, ATG2A, or ATG14 was evaluated using the chi-squared test. *P* < 0.05 was considered statistically significant.

## Supplementary information

Supplementary_Materials

Supplementary Table 1

Supplementary Table 2

Supplementary Table 3

Supplementary Table 4

Supplementary Table 5

## Data Availability

All data in our study are available upon request.
