# Supplementary material for: HIF-1α-induced expression of m6A reader YTHDF1 drives hypoxia-induced autophagy and malignancy of hepatocellular carcinoma by promoting ATG2A and ATG14 translation
Source: Signal Transduct Target Ther. 2021 Feb 23;6:76. doi: 10.1038/s41392-020-00453-8 (PMC7900110; doi:10.1038/s41392-020-00453-8)
Supplement: Supplementary file 1 — Supplementary_Materials [file 41392_2020_453_MOESM1_ESM.docx]

Supplementary Materials for

**HIF-1α-induced expression of m6A reader YTHDF1 drives hypoxia-induced autophagy and malignancy of hepatocellular carcinoma by promoting ATG2A and ATG14 translation**

Qing Li^1,2,†^· Runqiu Jiang^3,4,†^· Liren Zhang^1,†^· Hong Yang^5,†^· Jing Xu^6,†^· Yuanchang Hu^1^· Jiannan Qiu^1^· Yong Ni^7,*^· Liyong Pu^1,*^· Jinhai Tang^8,*^· Xuehao Wang^1,2^^,*^

^1^Hepatobiliary Center, The First Affiliated Hospital of Nanjing Medical University; Key Laboratory of Liver Transplantation, Chinese Academy of Medical Sciences; NHC Key Laboratory of Living Donor Liver Transplantation (Nanjing Medical University), Nanjing, Jiangsu Province, China.

^2^School of Medicine, Southeast University, Nanjing, China.

^3^Department of Hepatobiliary Surgery, The Affiliated Drum Tower Hospital of Nanjing University Medical School, Nanjing, Jiangsu Province, People's Republic of China.

^4^Medical School of Nanjing University, Nanjing, Jiangsu, China.

^5^Department of Immunology, Key Laboratory of Immune Microenvironment and Disease, Nanjing Medical University, Nanjing, Jiangsu Province, China.

^6^Department of Oncology, The First Affiliated Hospital of Nanjing Medical University, Nanjing, Jiangsu Province, China.

^7^Department of Hepatopancreatobiliary Surgery, Shenzhen Second People's Hospital, The First Affiliated Hospital of Shenzhen University, Shenzhen, Guangdong, China.

^8^Department of General Surgery, The First Affiliated Hospital of Nanjing Medical University, Nanjing, Jiangsu Province, China.

^†^These authors contributed equally to this work.

^*^**Correspondence to:** Xuehao Wang, **email:** [wangxh@njmu.edu.cn](mailto:wangxh@njmu.edu.cn)

Jinhai Tang, **email:** jhtang@njmu.edu.cn

Liyong Pu, **email:** [puliyong@njmu.edu.cn](mailto:puliyong@njmu.edu.cn)

Yong Ni, **email:** szniyong@sina.com

**This PDF file includes:**

Figures. S1 to S14

**Other Supplementary Materials for this manuscript include the following:**

Supplementary Table 1-5

Supplementary Table 1：Correlations between regulated m6A gene expression and autophagic flux in the HCC cell lines.

Supplementary Table 2：Correlations between YTHDF1 expression and clinical characteristics in HCC patients (n = 120).

Supplementary Table 3：Univariate and multivariate analysis of factors associated with overall survival and recurrence-free survival of 120 HCC patients.

Supplementary Table 4：Methylated RNA immunoprecipitation sequencing (MeRIP-seq) of hypoxic SMMC7721 cells.

Supplementary Table 5：Proteomics analysis of hypoxic SMMC7721 cells.


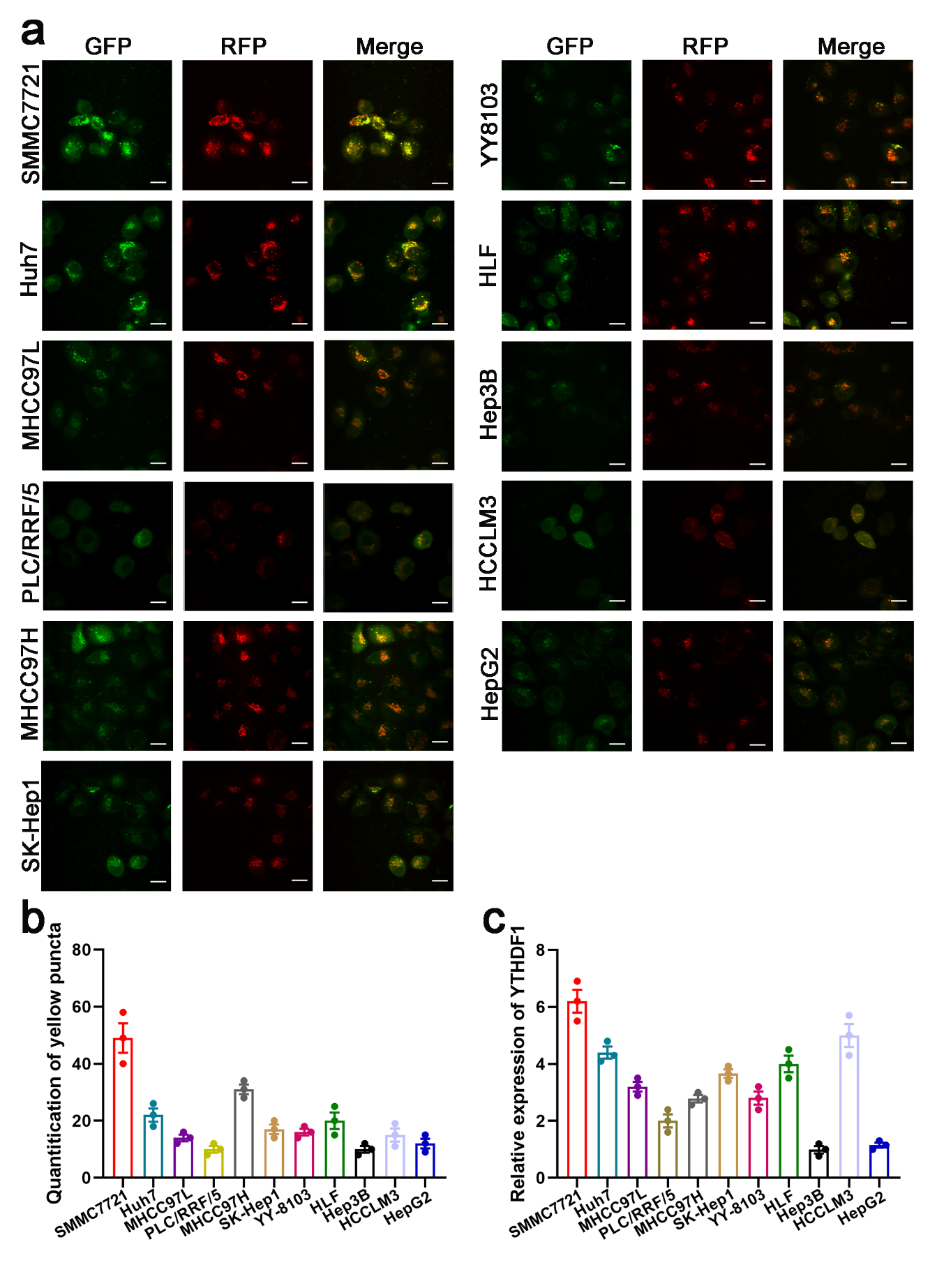
FigureS1

­**Figure S1. YTHDF1 is closely correlated with hypoxia-induced autophagy. a** Immunoﬂuorescence (IF) staining with mRFP-GFP-LC3 in HCC cell lines under hypoxia. Red puncta signify autolysosomes and yellow puncta signify autophagosomes. Scale bar, 10 µm. **b** Quantification of LC3 puncta under hypoxia. **c** YTHDF1 expression detected in HCC cell lines using qRT-PCR. Error bars represent the mean ± SEM and the dots represent the value of each experiment.


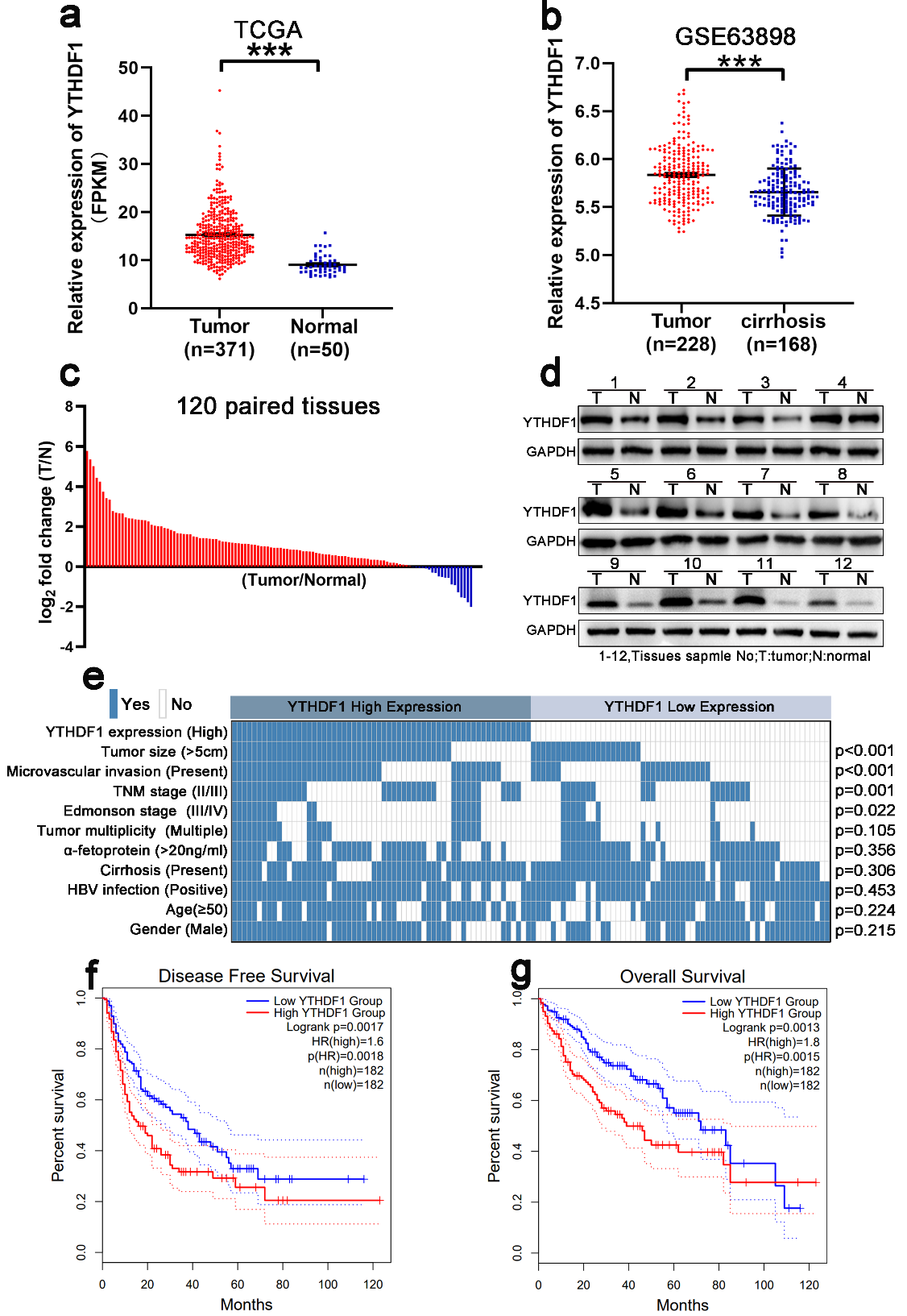
**FigureS2**

**Figure S2. YTHDF1 expression is increased in patients with HCC. a** Expression levels of YTHDF1 in HCC (n=371) and normal tissues (n=50) using the TCGA database. **b** Expression levels of YTHDF1 in HCC (n=228) and cirrhotic tissues (n=168) using the GEO database. **c** Relative expression of YTHDF1 using qRT-PCR in 120 paired HCC and corresponding adjacent non-tumorous tissues. **d** Protein levels of YTHDF1 in 12 HCC tissues and corresponding adjacent non-tumorous tissues using western blotting. **e** Correlations between YTHDF1 expression and clinical characteristics in patients with HCC (n=120). **f-g** Kaplan-Meier analysis demonstrating disease-free survival **(f)** and overall survival **(g)** of HCC patients with diverse YTHDF1 expression in the GEPIA database. Error bars represent the mean ± SEM and the dots represent the value of each experiment; ****P*<0.001.


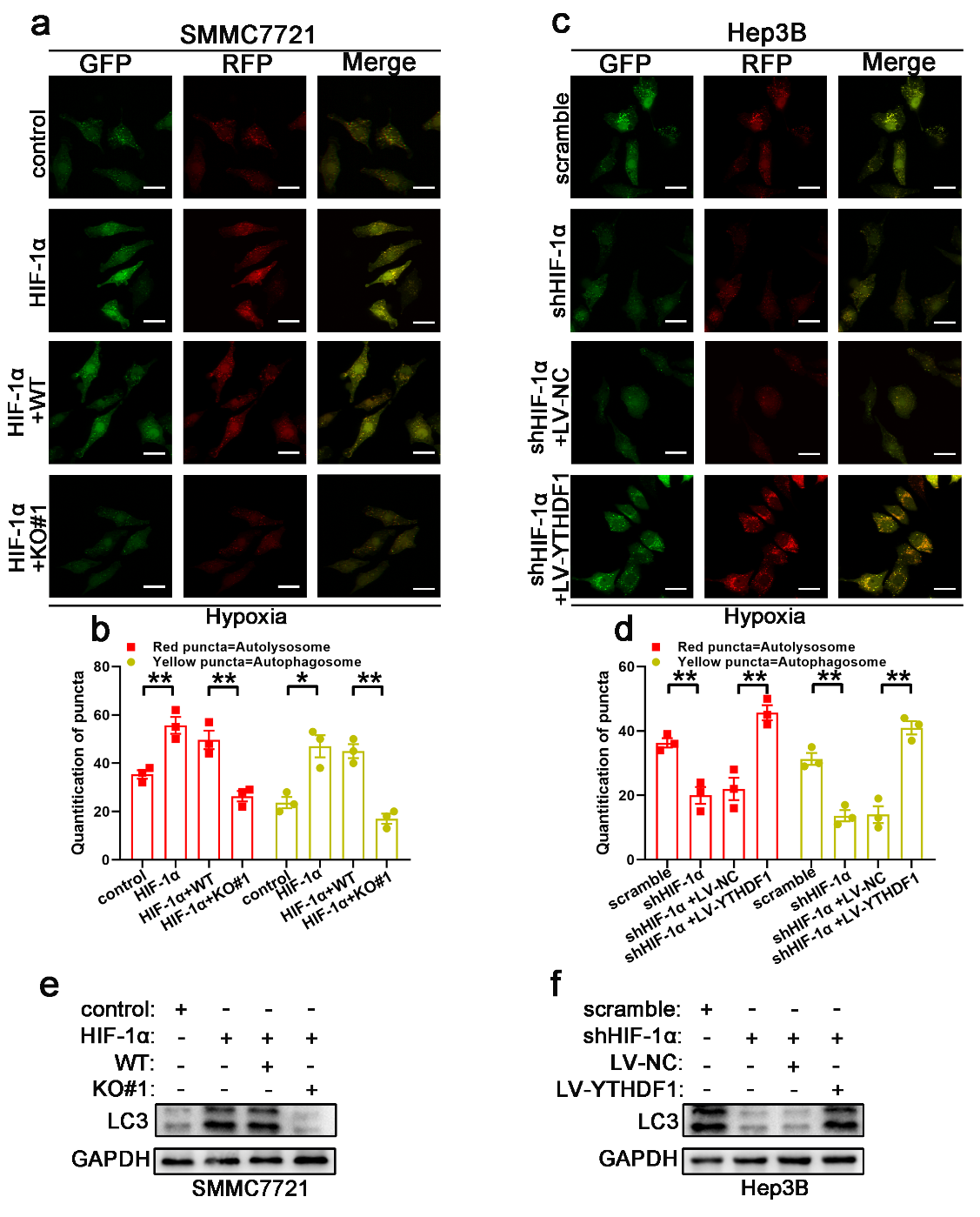
**FigureS3**

**Figure S3. HIF-1α/YTHDF1 signaling promotes hypoxia-induced autophagy.** **a** IF staining with mRFP-GFP-LC3 of hypoxic SMMC7721 cells in different groups. Red puncta signify autolysosomes and yellow puncta signify autophagosomes. Scale bar, 10 µm. **b** Quantification of LC3 puncta. **c** IF staining with mRFP-GFP-LC3 of hypoxic Hep3B cells in different groups. Scale bar, 10 µm. **d** Quantification of LC3 puncta. **e-f** Western blotting of LC3 expression in hypoxic SMMC7721 and Hep3B cells. Error bars represent the mean ± SEM and the dots represent the value of each experiment; **P*<0.05, ***P*<0.01.


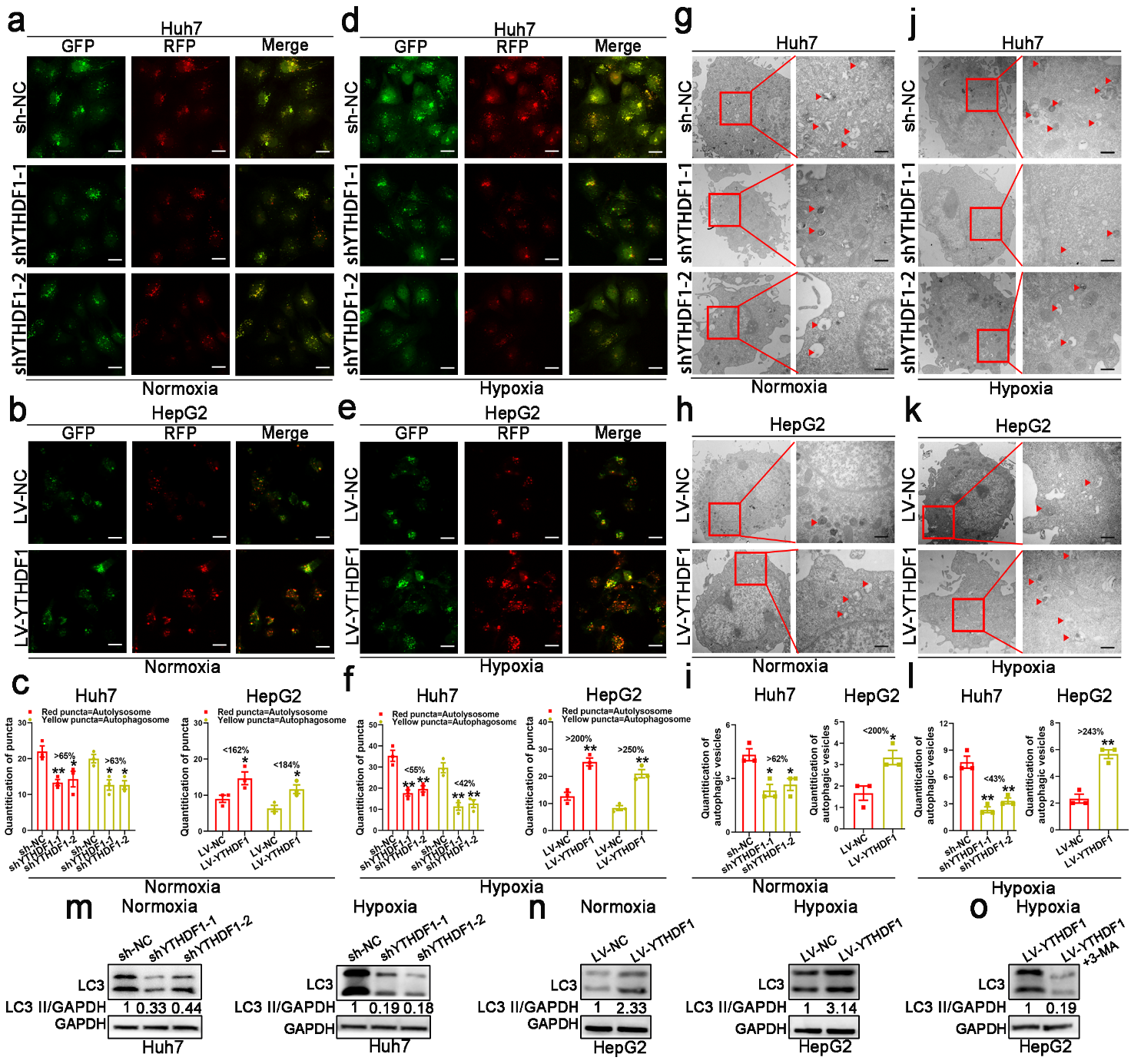
**FigureS4**

**Figure S4. YTHDF1 promotes hypoxia-induced autophagy in HCC cell lines. a-b** IF staining with mRFP-GFP-LC3 in normoxic Huh7 and HepG2 cells with YTHDF1 knockdown or overexpression, respectively. Red puncta signify autolysosomes and yellow puncta signify autophagosomes. Scale bar, 10 µm. **c** Quantification of LC3 puncta under normoxia. **d-e** IF staining with mRFP-GFP-LC3 in hypoxic Huh7 and HepG2 with YTHDF1 knockdown or overexpression, respectively. Red puncta signify autolysosomes and yellow puncta signify autophagosomes. Scale bar, 10 µm. **f** Quantification of LC3 puncta under hypoxia. **g-h** TEM demonstrating autolysosomes and autophagosomes in normoxic Huh7 and HepG2 cells with YTHDF1 knockdown or overexpression, respectively. Scale bar, 1 µm. **i** Quantification of autophagic vesicles under normoxia. **j-k** TEM demonstrating autolysosomes and autophagosomes in hypoxic Huh7 and HepG2 cells with YTHDF1 knockdown or overexpression, respectively. Scale bar, 1 µm. **l** Quantification of autophagic vesicles under hypoxia. **m** Western blotting demonstrating expression of LC3 in Huh7 cells with YTHDF1 knockdown under normoxia and hypoxia. **n** Western blotting demonstrating expression of LC3 in HepG2 cells with YTHDF1 overexpression under normoxia and hypoxia. **j** Western blotting demonstrating expression of LC3 in hypoxic YTHDF1 overexpressed HepG2 cells following 3-MA treatment. Error bars represent the mean ± SEM and the dots represent the value of each experiment; **P*<0.05, ***P*<0.01.


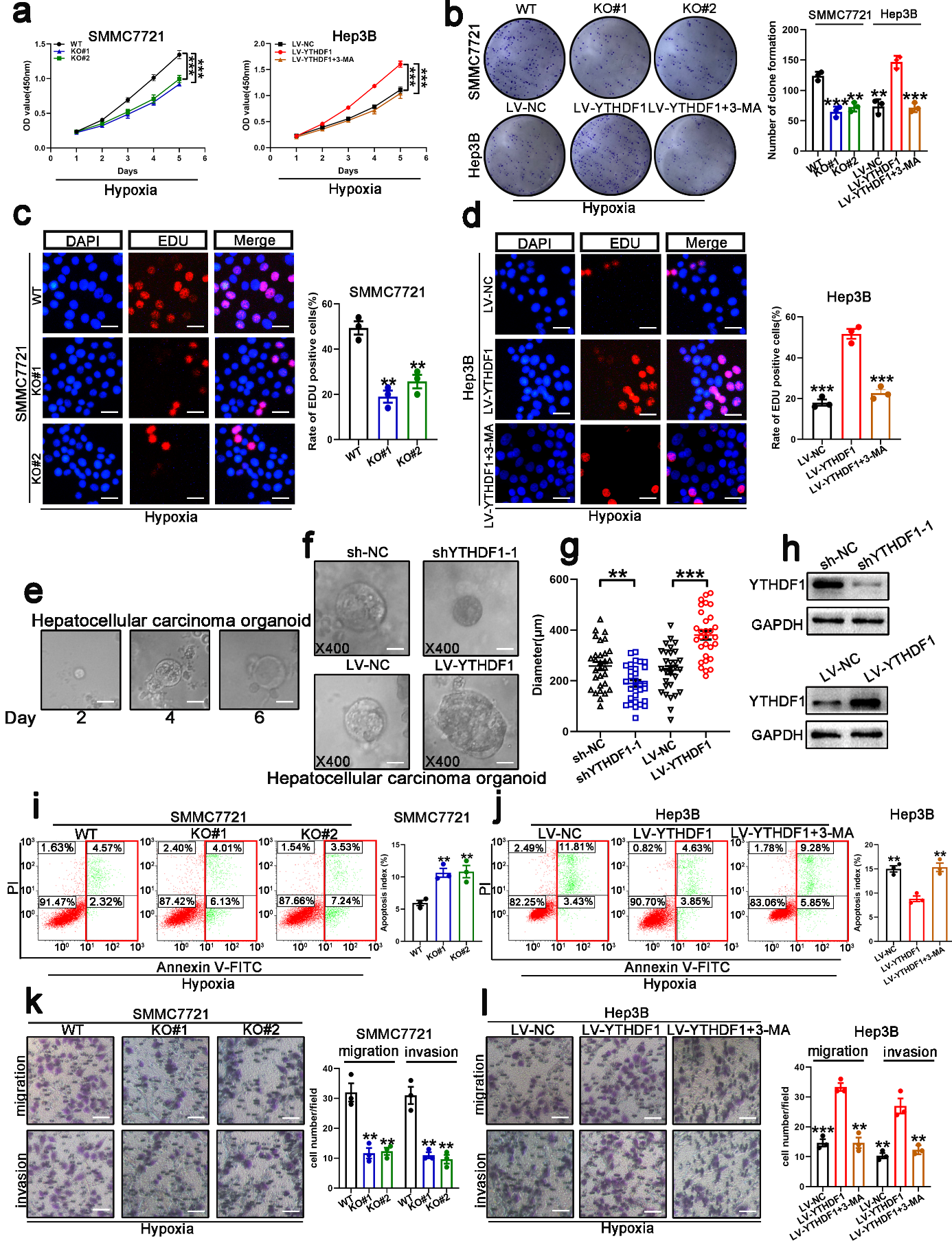
**FigureS5**

**Figure S5. YTHDF1 promotes autophagy-related malignancy in hypoxic SMMC7721 and Hep3B cell lines.** **a** CCK-8 assays in hypoxic SMMC7721 and Hep3B cells with YTHDF1 knockout or overexpression, respectively. 3-MA was used as an autophagy inhibitor. The OD value between KO#1 and KO#2 or LV-YTHDF1 and corresponding control was remarkably significant at P < 0.001 using two-way ANOVA. **b** Colony formation assays in hypoxic SMMC7721 and Hep3B cells with YTHDF1 knockout or overexpression, respectively. 3-MA was used as an autophagy inhibitor. **c-d** EdU assays in hypoxic SMMC7721 and Hep3B cells with YTHDF1 knockout or overexpression, respectively. 3-MA was used as an autophagy inhibitor. Scale bar, 50 µm. **e** Light field images of HCC tissue organoids (days 2, 4, and 6). Scale bar, 100 µm. **f** Light field images of HCC tissue organoids after YTHDF1 knockdown or overexpression mediated by lentivirus infection, or control lentivirus. Scale bar, 100 µm. **g** Diameters of HCC tissue organoids. **h** Protein levels of YTHDF1 in HCC tissue organoids using western blotting. **i-j** Cell apoptosis assays in hypoxic SMMC7721 and Hep3B cells with YTHDF1 knockout or overexpression, respectively. 3-MA was used as an autophagy inhibitor. **k-l** Migration and invasion assays in hypoxic SMMC7721 and Hep3B cells with YTHDF1 knockout or overexpression, respectively. 3-MA was used as an autophagy inhibitor. Scale bar, 50 µm. Error bars represent the mean ± SEM and the dots represent the value of each experiment; ***P*<0.01, ****P*<0.001.

**FigureS6**


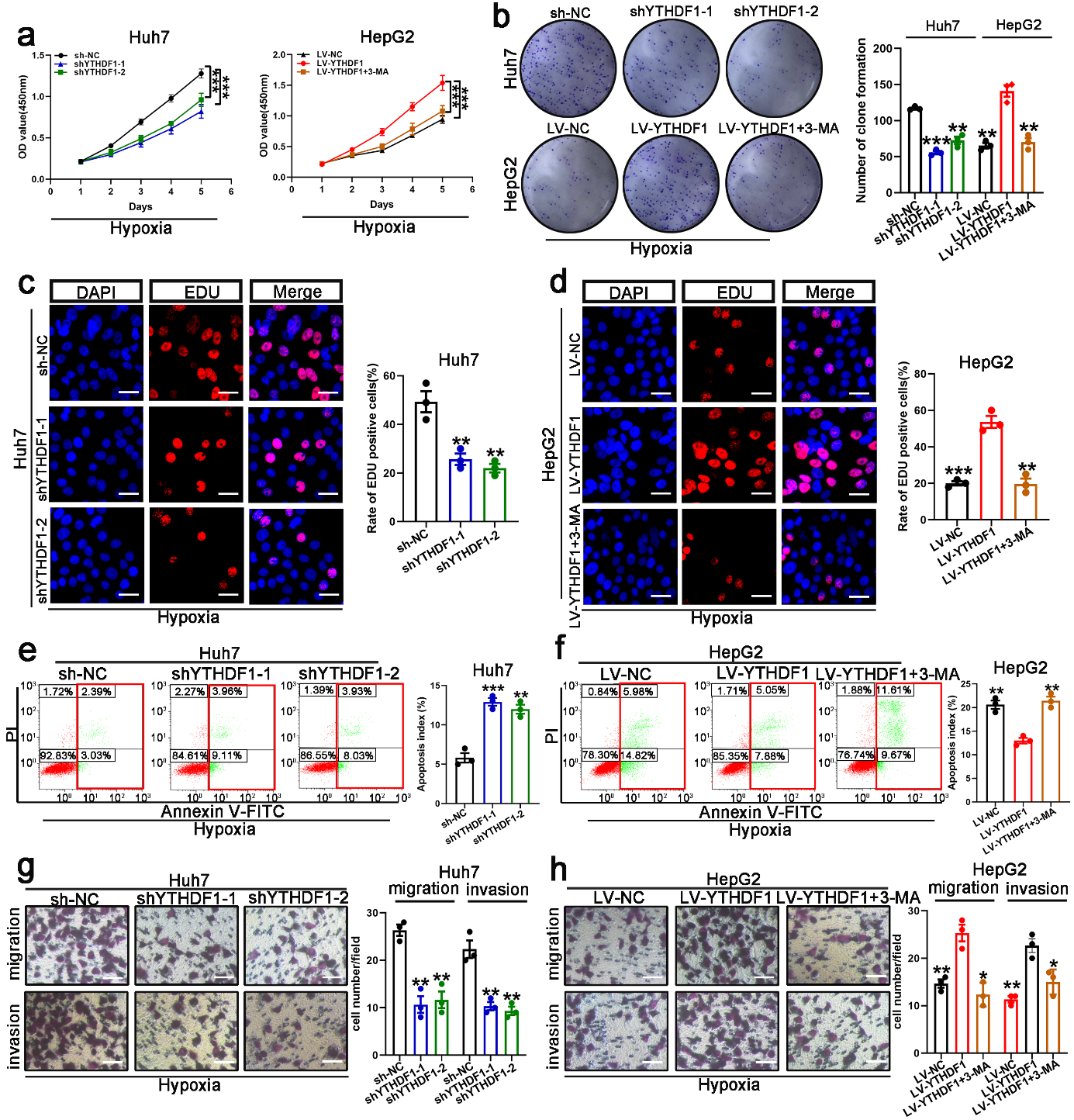


**Figure S6. YTHDF1 promotes autophagy-related malignancy in hypoxic Huh7 and HepG2 cell lines.** **a** CCK-8 assays in hypoxic Huh7 and HepG2 cells with YTHDF1 knockdown or overexpression, respectively. 3-MA was used as an autophagy inhibitor. The OD value between shYTHDF1-1 and shYTHDF1-2 or LV-YTHDF1 and the corresponding control was remarkably significant at P < 0.001 confirmed using two-way ANOVA. **b** Colony formation assays in hypoxic Huh7 and HepG2 cells with YTHDF1 knockdown or overexpression, respectively. 3-MA was used as an autophagy inhibitor. **c-d** EdU assays in hypoxic Huh7 and HepG2 cells with YTHDF1 knockdown or overexpression, respectively. 3-MA was used as an autophagy inhibitor. Scale bar, 50 µm. **e-f** Cell apoptosis assays in hypoxic Huh7 and HepG2 cells with YTHDF1 knockdown or overexpression, respectively. 3-MA was used as an autophagy inhibitor. **g-h** Migration and invasion assays in hypoxic Huh7 and HepG2 cells with YTHDF1 knockdown or overexpression, respectively. 3-MA was used as an autophagy inhibitor. Scale bar, 50 µm. Error bars represent the mean ± SEM and the dots represent the value of each experiment; **P*<0.05, ***P*<0.01, ****P*<0.001.


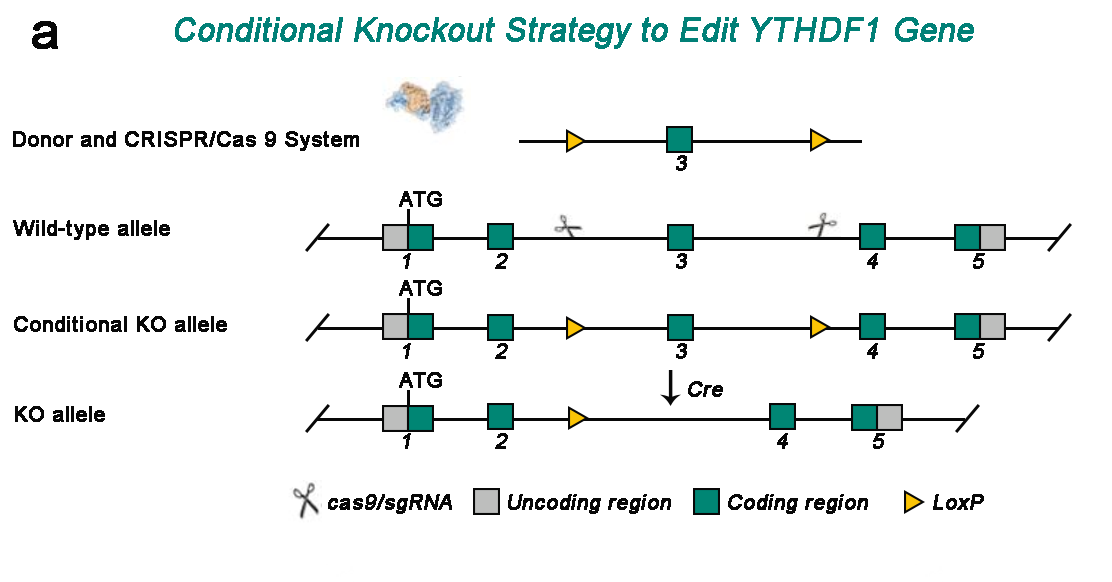
**FigureS7**

**Figure S7. Generation of liver-specific YTHDF1 knockout mice. a** Conditional knockout strategy to edit the YTHDF1 gene.


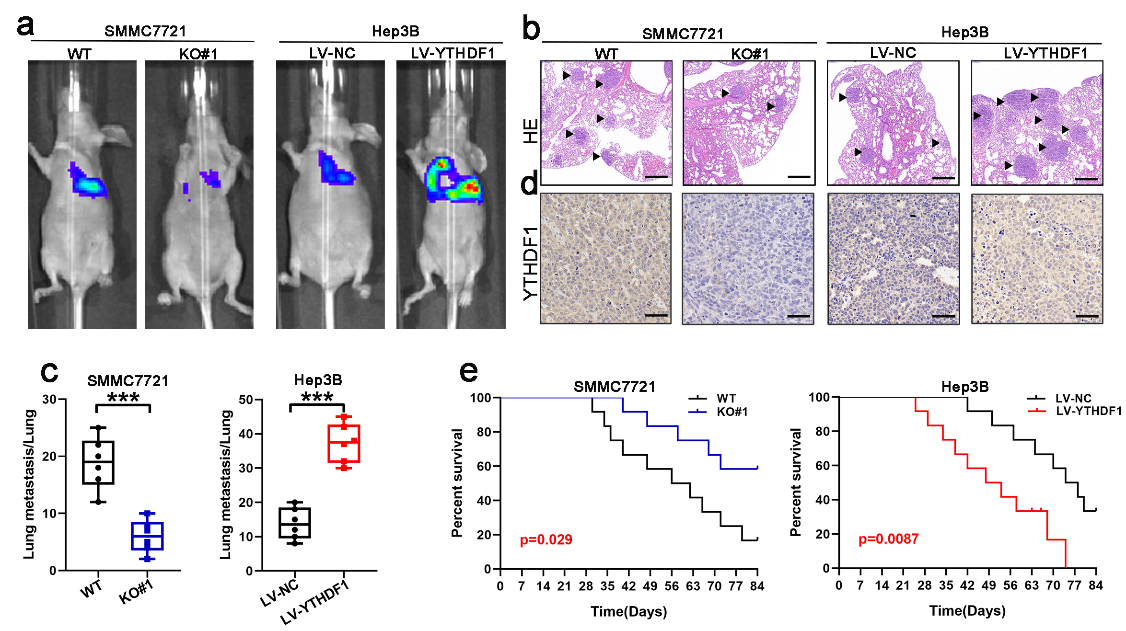
**FigureS8**

**Figure S8. YTHDF1** **facilitates HCC metastasis *in vivo*.** **a** Representative images of lung metastasis models in nude mice after tail vein injection of SMMC7721 cells and Hep3B cells with YTHDF1 knockout or overexpression, respectively. Fluorescence intensity was examined. **b** Hematoxylin and eosin (H&E) staining of pulmonary metastatic nodules. Scale bar, 200 µm. **c** Number of lung metastatic foci counted microscopically. **d** Representative immunohistochemistry (IHC) staining of YTHDF1 in lung metastatic nodules. Scale bar, 100 µm. **e** Overall survival of mice injected with SMMC7721 cells and Hep3B cells with YTHDF1 knockout or overexpression, respectively. The dots represent the value of each experiment; ****P*<0.001.

**FigureS9**


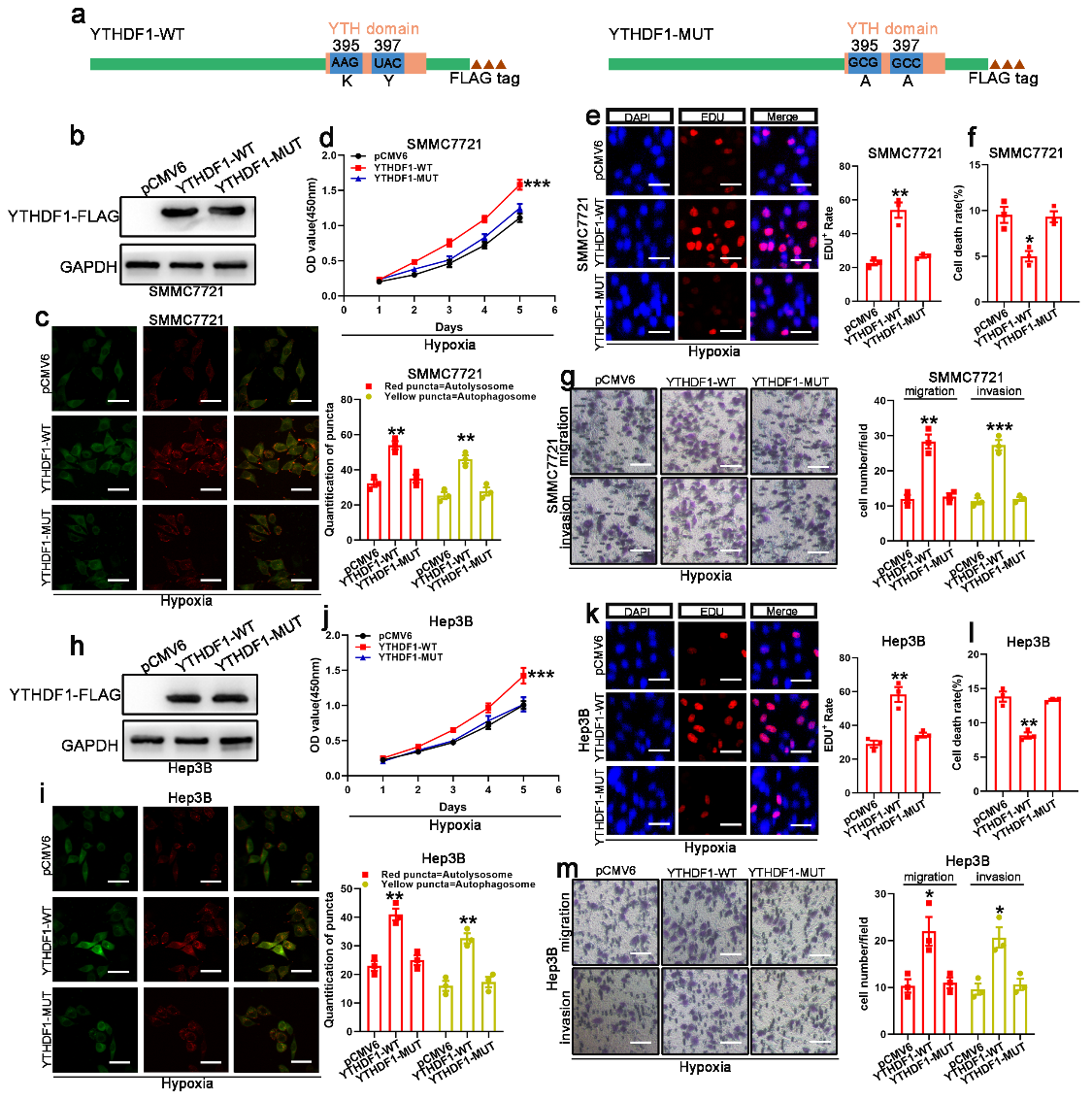


**Figure S9. m6A-binding pockets in the YTH domain are essential for YTHDF1 to play its oncogenic role.** **a** Schematic illustration of wild-type (YTHDF1-WT) and mutant (YTHDF1-MUT) YTHDF1 constructs. **b** YTHDF1 expression upon overexpression of YTHDF1-WT or YTHDF1-MUT in hypoxic SMMC7721 cells. **c** IF staining with mRFP-GFP-LC3 of hypoxic SMMC7721 cells with overexpressed YTHDF1-WT or YTHDF1-MUT. Scale bar, 10 µm. **d** CCK-8 assays of hypoxic SMMC7721 cells with overexpressed YTHDF1-WT or YTHDF1-MUT. **e** EDU assays of hypoxic SMMC7721 cells with overexpressed YTHDF1-WT or YTHDF1-MUT. Scale bar, 50 µm. **f** Cell apoptosis assays of hypoxic SMMC7721 cells with overexpressed YTHDF1-WT or YTHDF1-MUT. **g** Migration and invasion assays of hypoxic SMMC7721 cells with overexpressed YTHDF1-WT or YTHDF1-MUT. Scale bar, 50 µm. **h** YTHDF1 expression upon overexpression of YTHDF1-WT or YTHDF1-MUT in hypoxic Hep3B cells. **i** IF staining with mRFP-GFP-LC3 of hypoxic Hep3B cells with overexpressed YTHDF1-WT or YTHDF1-MUT. Scale bar, 10 µm. **j** CCK-8 assays of hypoxic Hep3B cells with overexpressed YTHDF1-WT or YTHDF1-MUT. **k** EDU assays of hypoxic Hep3B cells with overexpressed YTHDF1-WT or YTHDF1-MUT. Scale bar, 50 µm. **l** Cell apoptosis assays of hypoxic Hep3B cells with overexpressed YTHDF1-WT or YTHDF1-MUT. **m** Migration and invasion assays of hypoxic Hep3B cells with overexpressed YTHDF1-WT or YTHDF1-MUT. Scale bar, 50 µm. Error bars represent the mean ± SEM and the dots represent the value of each experiment; **P*<0.05, ***P*<0.01, ****P*<0.001.


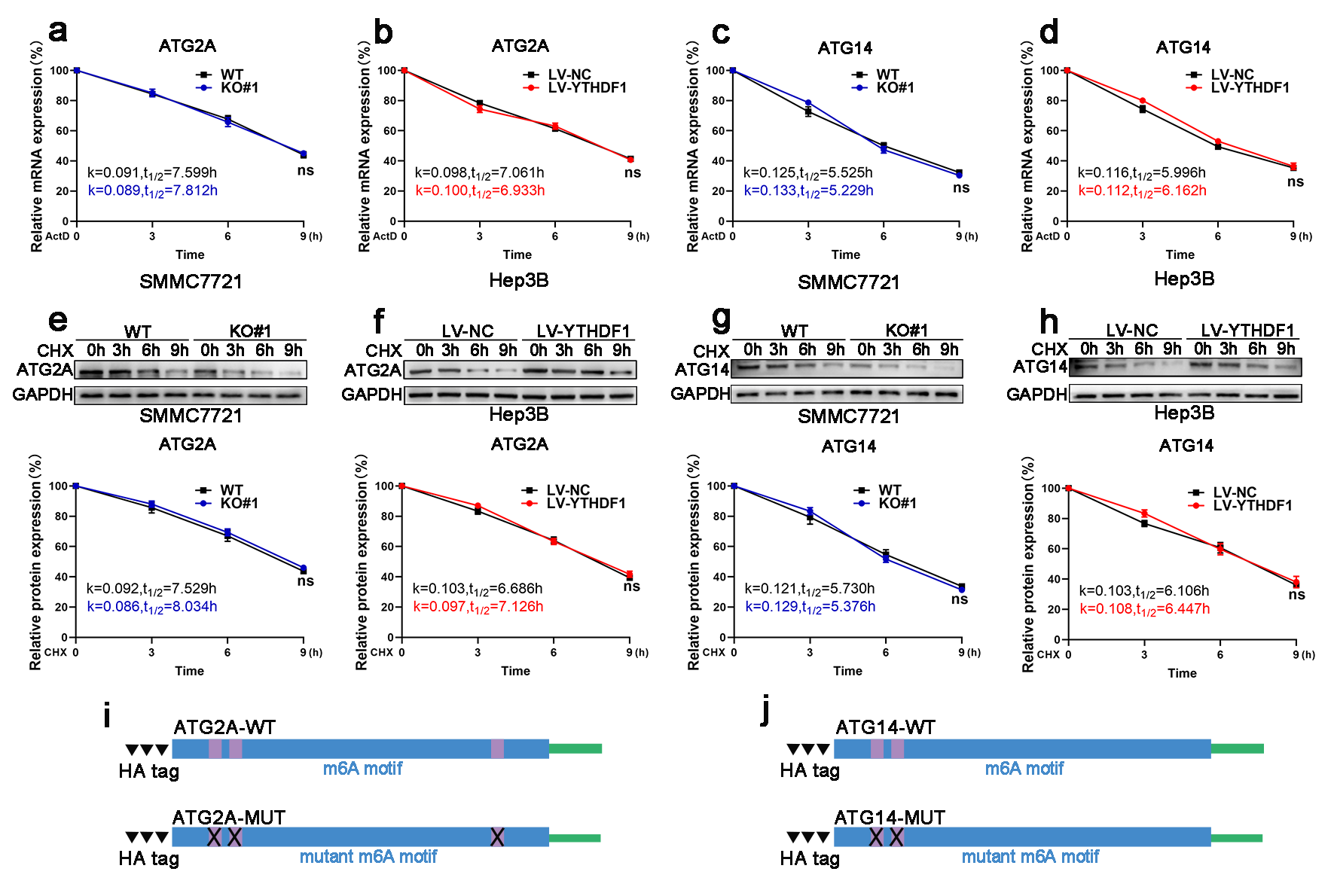
**FigureS10**

**Figure S10. YTHDF1 does not affect RNA stability and protein stability of ATG2A and ATG14. a-b** ATG2A mRNA expression upon actinomycin D treatment in hypoxic SMMC7721 and Hep3B cells. **c-d** ATG14 mRNA expression upon actinomycin D treatment in hypoxic SMMC7721 and Hep3B cells. **e-f** ATG2A protein expression upon cycloheximide (CHX) treatment in hypoxic SMMC7721 and Hep3B cells. **g-h** ATG14 protein expression upon CHX treatment in hypoxic SMMC7721 and Hep3B cells. **i** Schematic illustration of HA-tagged wild-type (ATG2A-WT) and mutant (ATG2A-MUT) ATG2A constructs. **j** Schematic illustration of HA-tagged wild-type (ATG14-WT) and mutant (ATG14-MUT) ATG14 constructs. ns, no significance.

**FigureS11**


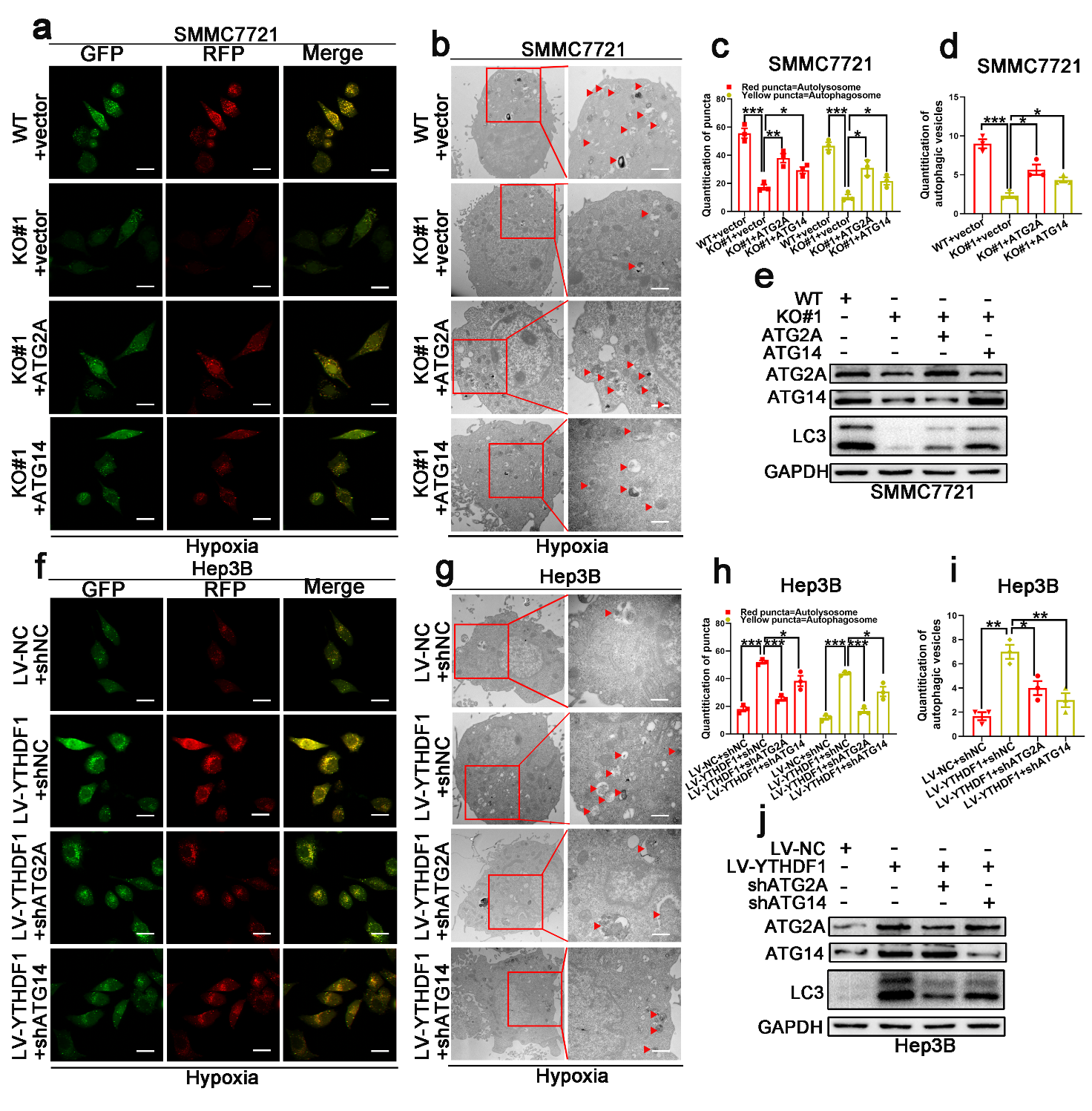


**Figure S11. YTHDF1 promotes hypoxia-induced autophagy dependent on ATG2A and ATG14 under hypoxia. a** IF staining with mRFP-GFP-LC3 detected by confocal microscopy. The decreased autophagy induced by YTHDF1-KO#1 was corrected by ATG2A and ATG14 overexpression in hypoxic SMMC7721 cells. Scale bar, 10 µm. **b** TEM showing autolysosomes and autophagosomes in hypoxic SMMC7721 cells. The decreased autophagy induced by YTHDF1-KO#1 was corrected by ATG2A and ATG14 overexpression. Scale bar, 1 µm. **c** Quantification of LC3 puncta in hypoxic SMMC7721 cells. **d** Quantification of autophagic vesicles. **e** Western blotting demonstrating expression of ATG2A, ATG14, and LC3 in hypoxic SMMC7721. **f** IF staining with mRFP-GFP-LC3 detected by confocal microscopy. The increased autophagy induced by LV-YTHDF1 was rescued by ATG2A and ATG14 knockdown. Scale bar, 10 µm. **g** TEM showing autolysosomes and autophagosomes in hypoxic Hep3B cells. The increased autophagy induced by LV-YTHDF1 was rescued by ATG2A and ATG14 knockdown. Scale bar, 1 µm. **h** Quantification of LC3 puncta in hypoxic Hep3B cells. **i** Quantification of autophagic vesicles in hypoxic Hep3B cells. **j** Western blotting showing expression of ATG2A, ATG14, and LC3 in hypoxic Hep3B cells. Error bars represent the mean ± SEM and the dots represent the value of each experiment; **P*<0.05, ***P*<0.01, ****P*<0.001.

**FigureS12**


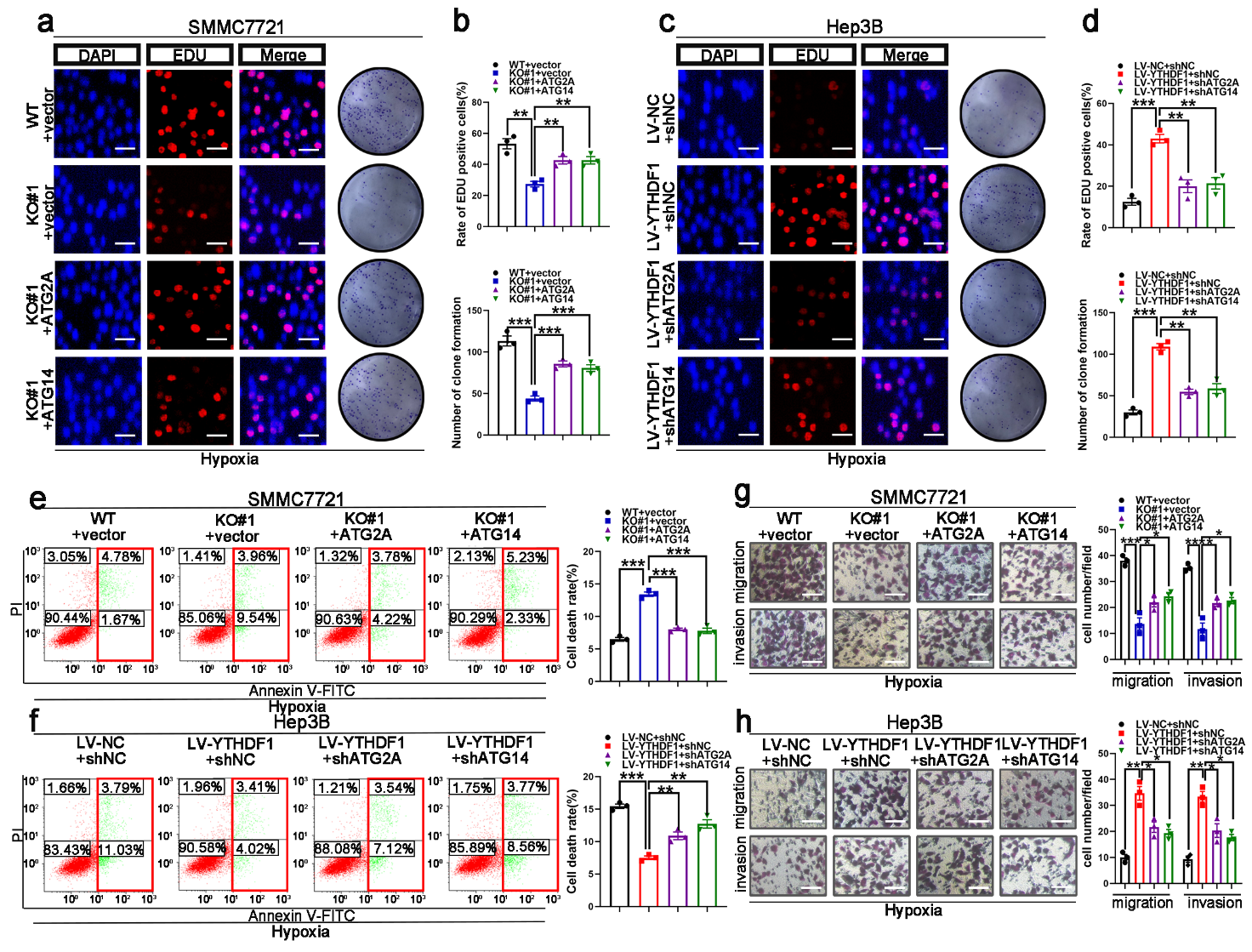
**­**

**Figure S12. YTHDF1 promotes autophagy-related malignancy dependent on ATG2A and ATG14 under hypoxia. a-b** EdU and colony formation assays demonstrating the impaired proliferation ability induced by YTHDF1-KO#1 was corrected by ATG2A and ATG14 overexpression in hypoxic SMMC7721 cells. Scale bar, 50 µm. **c-d** EdU and colony formation assays demonstrating the increased proliferation ability induced by LV-YTHDF1 was rescued by ATG2A and ATG14 knockdown. Scale bar, 50 µm. **e** Cell apoptosis assays demonstrating the increased apoptosis ability induced by YTHDF1-KO#1 was corrected by ATG2A and ATG14 overexpression in hypoxic SMMC7721 cells. **f** Cell apoptosis assays demonstrating the decreased apoptosis ability induced by LV-YTHDF1 was rescued by ATG2A and ATG14 knockdown. **g** Migration and invasion assays demonstrating the impaired migration and invasion induced by YTHDF1-KO#1 was corrected by ATG2A and ATG14 overexpression in hypoxic SMMC7721 cells. Scale bar, 50 µm. **h** Migration and invasion assays demonstrating the increased migration and invasion induced by LV-YTHDF1 could be rescued by ATG2A and ATG14 knockdown. Scale bar, 50 µm. Error bars represent the mean ± SEM and the dots represent the value of each experiment; **P*<0.05, ***P*<0.01, ****P*<0.001.


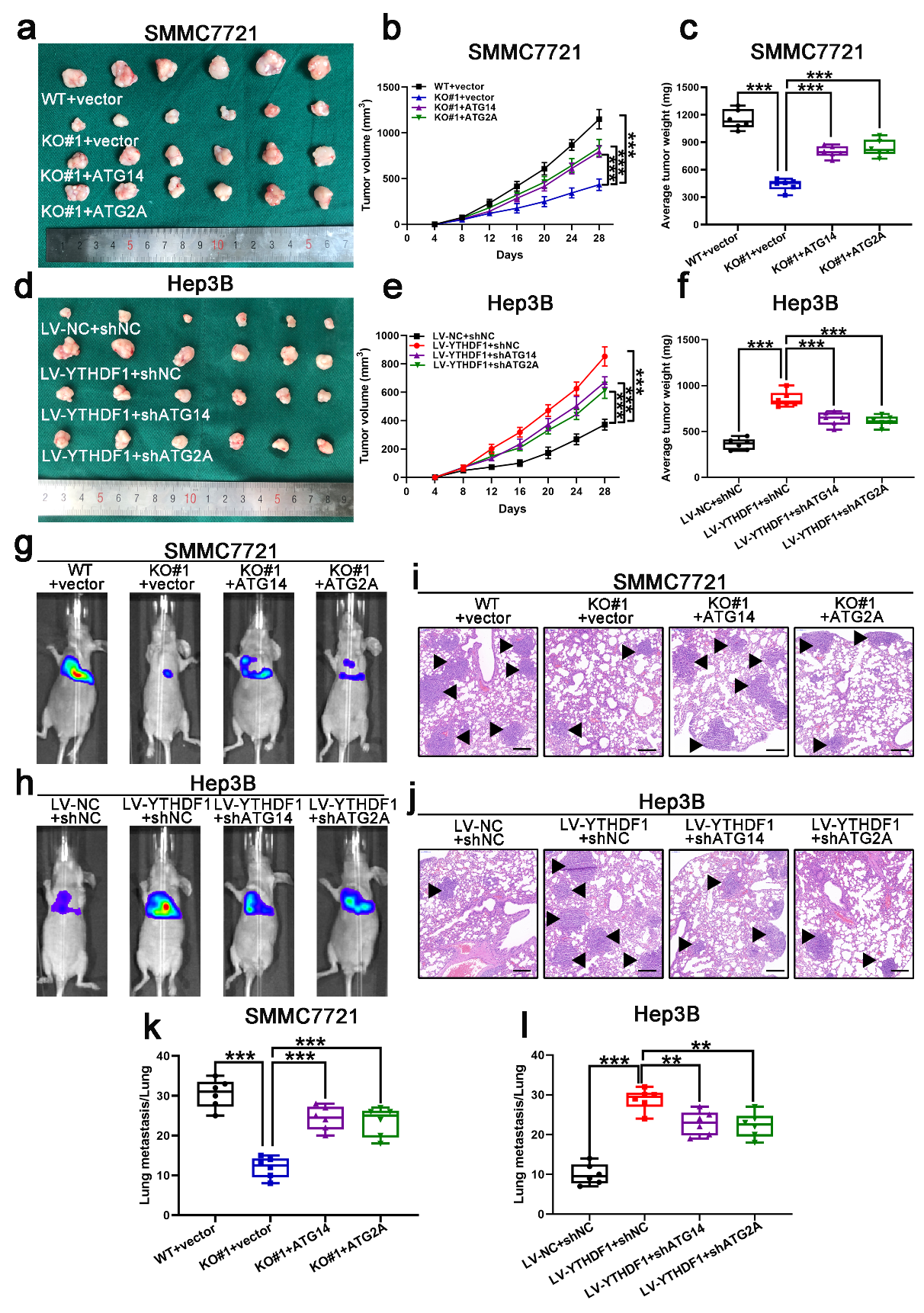
**FigureS13**

**Figure S13. ATG2A and ATG14 are functionally targeted genes of YTHDF1 in vivo.** **a-c** Representative images of tumors (a), tumor volumes (b), and tumor weights (c) in nude mice bearing SMMC7721 cells in different groups. **d-f** Representative images of tumors (d), tumor volumes (e), and tumor weights (f) in nude mice bearing Hep3B cells in different groups. **g** Representative bioluminescence imaging of lung metastasis in nude mice bearing SMMC7721 cells in different groups. **h** Representative bioluminescence imaging of lung metastasis in nude mice bearing Hep3B cells in different groups. **i** H&E staining of lung tissues bearing SMMC7721 cells in different groups. Scale bar, 200 µm. **j** H&E staining of lung tissues bearing Hep3B cells in different groups. Scale bar, 200 µm. **k-l** Quantification of lung metastatic foci. Error bars represent the mean ± SEM and the dots represent the value of each experiment; ***P*<0.01, ****P*<0.001.


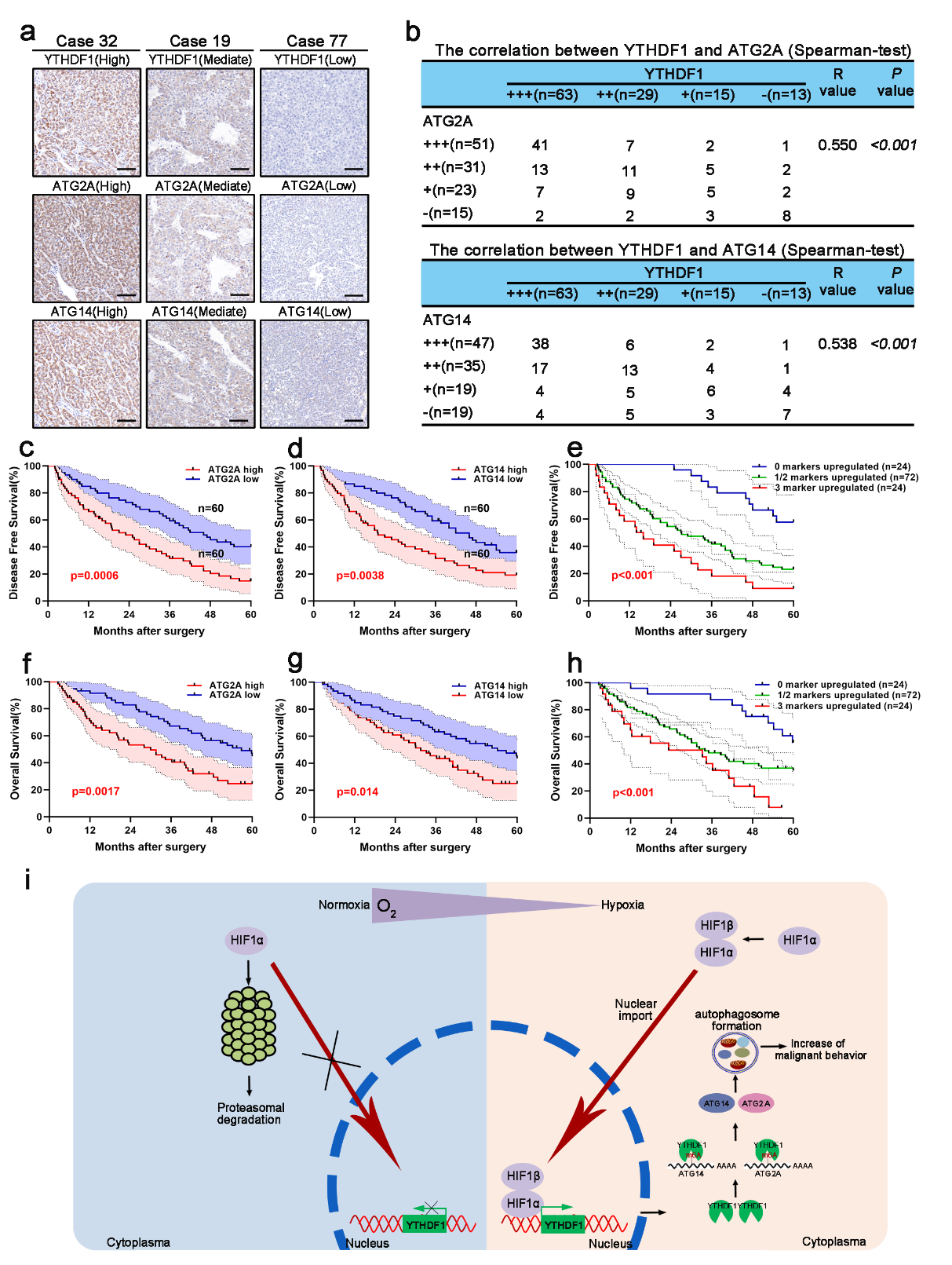
**FigureS14**

**Figure S14. Levels of YTHDF1 and ATG2A and ATG14 are clinically relevant in HCC patients. a** Representative IHC images of YTHDF1, ATG2A, and ATG14 in HCC tissues. Scale bar, 100 µm. **b** Spearman’s correlation between YTHDF1 expression and ATG2A IHC scores (upper). Spearman’s correlation between YTHDF1 expression and ATG14 IHC scores (lower). **c-d** Kaplan-Meier analysis showing the disease-free survival of HCC patients with diverse ATG2A **(c)** or ATG14 **(d)** expression. **e** Kaplan–Meier analysis of disease-free survival for HCC patients based on the number of upregulated molecular markers (YTHDF1, ATG2A, and ATG14). **f-g** Kaplan-Meier analysis demonstrating the overall survival of HCC patients with diverse ATG2A **(f)** or ATG14 **(g)** expression. **h** Kaplan–Meier analysis of overall survival for HCC patients based on the number of upregulated molecular markers (YTHDF1, ATG2A, and ATG14). **i** Proposed model underlying the roles of YTHDF1-mediated ATG2A or ATG14 translation and hypoxia-induced autophagy in HCC.
