# Supplementary material for: HIF-1α-induced expression of m6A reader YTHDF1 drives hypoxia-induced autophagy and malignancy of hepatocellular carcinoma by promoting ATG2A and ATG14 translation
Source: Signal Transduct Target Ther. 2021 Feb 23;6:76. doi: 10.1038/s41392-020-00453-8 (PMC7900110; doi:10.1038/s41392-020-00453-8)
Supplement: Supplementary file 3 — Supplementary Table 2 [file 41392_2020_453_MOESM3_ESM.doc]

| **Supplementary Table 2** Correlations between YTHDF1 expression and clinical characteristics in HCC patients (n = 120) | | | | |
| --- | --- | --- | --- | --- |
| Characteristics | Number(%) | YTHDF1 expression | | P-value |
|  |  | Low group | High group |  |
| Age(years) |  |  |  |  |
| <50 | 34(28.3%) | 20 | 14 | 0.224 |
| ≥50 | 86(71.7%) | 40 | 46 |  |
| Gender |  |  |  |  |
| FeMale | 32(26.7%) | 19 | 13 | 0.215 |
| Male | 88(73.3%) | 41 | 47 |  |
| Cirrhosis  Present  Absent  HBV infection  Positive  Negative  Tumor size(cm) | 102(85.0%)  18(15.0%)  101(84.2%)  19(15.8%) | 53  7  52  8 | 49  11  49  11 | 0.306  0.453 |
| <5 | 54(45.0%) | 38 | 16 | **<0.001**^#^ |
| ≥5 | 66(55.0%) | 22 | 44 |  |
| Microvascular invasion  Present  Absent | 60(50.0%)  60(50.0%) | 20  40 | 40  20 | **<0.001**^#^ |
| Tumor multiplicity  Simple  Multiple | 86(71.7%)  34(28.3%) | 47  13 | 39  21 | 0.105 |
| α-fetoprotein (ng/ml)  ≤20  >20  TNM stage | 51(42.5%)  69(57.5%) | 28  32 | 23  37 | 0.356 |
| I | 66(55.0%) | 42 | 24 | **0.001^**^** |
| II/III | 54(45.0%) | 18 | 36 |  |
| Edmonson stage |  |  |  |  |
| I/II | 96(80.0%) | 53 | 43 | **0.022^*^** |
| III/IV | 24(20.0%) | 7 | 17 |  |

| *P<0.05 , **P<0.01 , ^#^P<0.001 |
| --- |
|  |
