# Supplementary material for: HIF-1α-induced expression of m6A reader YTHDF1 drives hypoxia-induced autophagy and malignancy of hepatocellular carcinoma by promoting ATG2A and ATG14 translation
Source: Signal Transduct Target Ther. 2021 Feb 23;6:76. doi: 10.1038/s41392-020-00453-8 (PMC7900110; doi:10.1038/s41392-020-00453-8)
Supplement: Supplementary file 4 — Supplementary Table 3 [file 41392_2020_453_MOESM4_ESM.doc]

**Supplementary Table 3** Univariate and multivariate analysis of factors associated with overall survival and recurrence-free survival of 120 HCC patients

|  | |  | Recurrence-free survival Overall survival | | | | | | |
| --- | --- | --- | --- | --- | --- | --- | --- | --- | --- |
| Clinicopathologic Parameters |  | Univariate | Multivariate analysis | | | Univariate | Multivariate analysis | | |
|  | | *P* value | HR | 95% CI | *P* value | *P* value | HR | 95% CI | *P* value |
| Age (>50 years vs ≤50 years)  Gender (female vs male)  Cirrhosis (present vs absent)  HBV infection (positive vs negative)  TNM stage (II/III vs I)  Microvascular invasion (yes vs no)  Tumor multiplicity (multiple vs simple)  α-fetoprotein (≥20ng/ml vs <20ng/ml)  Edmonson stage (III/IV vs I/II)  Tumor size (≥5cm vs <5cm)  YTHDF1 expression ( high vs low)^a^ | | 0.658 |  |  |  | 0.569 |  |  |  |
|  |  | 0.124 |  |  |  | 0.099 |  |  |  |
|  |  | 0.282 |  |  |  | 0.244 |  |  | |
|  |  | 0.748 |  |  |  | 0.737 |  |  |  |
|  |  | 0.119 |  |  |  | 0.077 |  | | |
|  |  | **<0.001***** | 2.076 | 1.279-3.369 | **0.003**** | **0.007^***^** | 1.684 | 1.003-2.828 | **0.049*** |
|  |  | 0.273 |  |  |  | 0.261 |  | | |
|  |  | **0.029**** | 1.985 | 1.123-2.957 | **0.015*** | **0.013^*^** | 1.887 | 1.113-3.200 | **0.018*** |
|  |  | 0.213 |  |  |  | 0.329 |  | | |
|  |  | **0.023*** | NI |  |  | **0.009^***^** | NI |  |  |
|  |  | **<0.001***** | 1.822 | 1.030-2.859 | **0.038*** | **<0.001^***^** | 1.824 | 1.041-3.194 | **0.036*** |

*P < 0.05, ** P < 0.01, *** P < 0.001

^a^Using median YTHDF1 values as cutoff

HR, hazard ratio; CI, confidence interval; HBV, hepatitis B virus; TNM, tumor-node-metastasis; NI, not included.

The bold number means statistically significant.
